# Cerebrolysin Ameliorates Focal Cerebral Ischemia Injury Through Neuroinflammatory Inhibition *via* CREB/PGC-1α Pathway

**DOI:** 10.3389/fphar.2019.01245

**Published:** 2019-10-22

**Authors:** Xin Guan, Yunjie Wang, Guoyin Kai, Shunyi Zhao, Tingyu Huang, Youzhen Li, Yuan Xu, Luyong Zhang, Tao Pang

**Affiliations:** ^1^Jiangsu Key Laboratory of Drug Screening, State Key Laboratory of Natural Medicines, China Pharmaceutical University, Nanjing, China; ^2^College of Pharmaceutical Science, Zhejiang Chinese Medical University, Hangzhou, China; ^3^Guangdong Long Fu Pharmaceutical Co., Ltd., Zhongshan, China; ^4^School of Medicine and Life Sciences, Nanjing University of Chinese Medicine, Nanjing, China

**Keywords:** cerebrolysin, ischemic stroke, microglia, cAMP response element-binding protein, peroxisome proliferator-activated receptor gamma co-activator 1α

## Abstract

Neuroinflammation is one of the important factors aggravating brain injury after ischemic stroke. We aimed to investigate the effects of cerebrolysin (CBL) on neuroinflammation *in vivo* and *in vitro* and the underlying mechanisms. The gene expressions of pro-inflammatory factors and anti-inflammatory factors were analyzed by real time PCR in rat transient middle cerebral artery occlusion (tMCAO) model, lipopolysaccharides-induced neuroinflammatory mice model and LPS-treated mouse primary microglia cells. The neuroprotective effects of CBL were evaluated by infarct size, Longa test and Rotarod test for long-term functional recovery in rats subjected to ischemia. The role of CREB/PGC-1α pathway in anti-neuroinflammatory effect of CBL was also determined by real time PCR and Western blotting. In the tMCAO model, administration of CBL at 3 h post-ischemia reduced infarct volume, promoted long-term functional recovery, decreased the gene expression of pro-inflammatory factors and increased the gene expression of anti-inflammatory factors. Correspondingly, in LPS-induced neuroinflammatory mice model, CBL treatment attenuated sickness behavior, decreased the gene expression of pro-inflammatory factors, and increased the gene expression of anti-inflammatory factors. In *in vitro* and *in vivo* experiments, CBL increased the protein expression levels of PGC-1α and phosphorylated CREB to play anti-inflammatory effect. Additionally, the application of the specific CREB inhibitor, 666-15 compound could effectively reverse the anti-inflammatory effect of CBL in primary mouse microglia cells and anti-ischemic brain injury of CBL in rats subjected to tMCAO. In conclusion, CBL ameliorated cerebral ischemia injury through reducing neuroinflammation partly *via* the activation of CREB/PGC-1α pathway and may play a therapeutic role as anti-neuroinflammatory agents in the brain disorders associated with neuroinflammation.

## Introduction

Ischemic stroke is a major type of stroke causing brain injury without effective treatment worldwide. According to statistics, the mortality rate of ischemic stroke patients is 157 per 100,000 in China (Zhou et al., 2014). Currently, the only clinic-approved drug for the treatment of ischemic stroke is recombinant tissue plasminogen activator (r-tPA), but it has low clinical effect due to short time window and side effects (Snow, 2017).

Cerebrolysin (CBL) is a neuropeptides preparation with a complex mixture of peptides and amino acids, which act as neurotrophic factors. A meta-analysis of nine randomized clinical trials showed that CBL has a beneficial effect on early global neurological deficits in patients with acute ischemic stroke and safety aspects comparable to placebo (Bornstein et al., 2018). In animal model, previous studies have indicated that CBL administration could help improve neurological recovery, promote neurite outgrowth, resulting in treating acute ischemia stroke, traumatic brain injury, subarachnoid hemorrhage, and Alzheimer’s disease (Ubhi et al., 2013; Zhang et al., 2015; Park et al., 2018; Sadigh-Eteghad et al., 2018). The previous study by Barakat et al. (2014) found that CBL pretreatment before ischemia improves stroke functional and histological outcomes through reduced cytokine and immune cell infiltration within the ischemic hemisphere. However, whether CBL administrated after ischemia also has beneficial effects on ischemic brain injury and neurological deficits with detailed molecular mechanisms needs to be determined. Investigating the behavioral functional recovery and underlying mechanisms of CBL is beneficial for discovery of a series of drugs for the treatment of stroke.

Microglia are the resident macrophages in the brain. Once the ischemic stroke occurs, microglia can be rapidly activated during the early phase of ischemia (Ma et al., 2017). On the one hand, the pro-inflammatory phenotype of microglia can produce a variety of pro-inflammatory cytokines (IL-1β, IL-6, TNF-α, CCL2, and CXCL10) (Smith et al., 2012), ROS and NO to exacerbate brain injury. On the other hand, the anti-inflammatory phenotype, also called alternatively activated microglia, enhances the expression of Arginase 1, YM 1/2, IGF-1, and CD206 (Cunningham, 2013), contributing to tissue repair and remodeling. Therefore, promoting the switch of microglia toward a neuroprotective anti-inflammatory phenotype might be a promising approach for ischemic stroke therapy.

CREB, cyclic adenosine monophosphate (cAMP) response element binding protein, has been proved as a transcription factor that participated in many physiological processes, including proliferation, differentiation, and survival (Kandel, 2012). CREB can also regulate inflammation. The phosphorylation of CREB can directly block the binding to nuclear factor-κ-gene binding (NF-κB) complex, which can significantly reduce NF-κB activation (Wen et al., 2010). CREB is also an important transcription factor regulating the expression of peroxisome proliferator-activated receptor gamma co-activator 1α (PGC-1α) (Raefsky and Mattson, 2017). PGC-1α is a transcriptional coactivator that recruits nuclear receptors or transcription factors, and regulates transcription of downstream genes in both the nucleus and the mitochondria (Hursting and Dunlap, 2012; Mou et al., 2015; Wang et al., 2018). Instead of binding directly to nuclear or mitochondrial DNA, PGC-1α localizes in complexes containing several other proteins. PGC-1α is known to independently combat ROS production, inhibiting the pathway of NF-κB (Sheng et al., 2012), thereby reducing the occurrence of inflammation (Eisele et al., 2013).

In the present study, we sought to investigate whether CBL could reduce neuroinflammation to ameliorate brain injury in cerebral ischemic stroke model and LPS-induced neuroinflammatory model and further elucidate the possibly involved mechanisms.

## Materials and Methods

### Animals

All male Sprague Dawley rats (weight 260–280 g) were purchased from Zhejiang Laboratory Animals Center (Hangzhou, China). Male C57BL/6 (25–30 g) mice were purchased from Comparative Medicine Centre (Yangzhou University, China). All experiments carried out in accordance with the Guide for the Care and Use of Laboratory Animals of the National Institute of Health. Animals used were approved by the Committee of Experimental Animals in Jiangsu Province and the Ethics Committee of China Pharmaceutical University.

### Animal Experiments Design

Total of three separate animal experiments were carried out as shown in **Supplement Figures 1A, B and C**.

#### Experiment 1

*Experiment 1* was carried out to evaluate the effects of CBL on ameliorating ischemic area in rats after stroke and related neuroinflammatory factors as well as the underlying mechanisms. A total of 120 rats were divided into six groups: Sham, Stroke, Stroke+CBL (10 mg/kg) at 3 and 24 h after ischemia, Stroke+CBL (60 mg/kg) at 3 and 24 h after ischemia, Stroke+CBL (60 mg/kg) at 6 and 24 h after ischemia, and Stroke+CBL (60 mg/kg)+666-15 (10 mg/kg) at 3 and 24 h after ischemia (**Figure S1A**).

#### Experiment 2

*Experiment 2* was carried out to measure the effect of CBL on long-term functional recovery in rats after stroke. A total of 90 rats were divided into three groups: Sham, Stroke group at 3 and 24 h after ischemia, and Stroke+CBL (60 mg/kg) at 3 h and 24 h after ischemia (**Figure S1B**).

#### Experiment 3

*Experiment 3* was to measure the effect of CBL on LPS-induced neuroinflammatory mice model. A total of 80 C57BL/6 mice were divided into five groups: Control group, LPS (0.33 mg/kg), LPS (0.33 mg/kg)+CBL (20 mg/kg), LPS (0.33 mg/kg)+CBL (60 mg/kg), and LPS+CBL (100 mg/kg) (**Figure S1C**).

### Materials

CBL was provided by Guangdong Long Fu Pharmaceutical Co., Ltd. (Zhongshan, China). Rabbit anti-phospho-CREB (1:1,000), anti-CREB (1:1,000), anti-PGC-1α (1:1,000), anti-β-actin (1:10,000) antibodies were purchased from the ABclonal company. Rabbit anti-phospho-ERK1/2 (1:1,000), anti-ERK1/2 (1:1,000), anti-phospho-JNK (1:1,000), anti-JNK (1:1,000), anti-phospho-p38 MAPK (1:1,000), anti-p38 MAPK (1:1,000) antibodies were purchased from Cell Signaling Technology (Beverly, MA). Compound 666-15, the inhibitor of CREB was purchased from MedChemExpress (MCE, Shanghai, China). RIPA lysis buffer and LDH kit were purchased from Beyotime Biotechnology (Nanjing, China). Trizol reagent and the cDNA synthesis kit were purchased from Vazyme (Nanjing, China). SYBR Green was purchased from Invitrogen (Camarillo, CA). LPS was purchased from Sigma-Aldrich (St. Louis, USA). Cell culture medium and supplements were purchased from Invitrogen (Carlsbad, CA, USA). TTC (2, 3, 5-triphenyltetrazolium chloride) was bought from Sigma-Aldrich.

### Transient Middle Cerebral Artery Occlusion (tMCAO) and Drug Treatment

The healthy male SD rats were randomly divided into a series of groups for transient middle cerebral artery occlusion (tMCAO) (n = 12–15 for each group of successfully treated rats). Firstly, the rats were treated with anesthesia in an isoflurane chamber with 3.5% isoflurane, and then 2% isoflurane was maintained through a mask in the operation. During the surgery, the animals were placed on a heating device to ensure normal body temperature (37°C). After accurate separation of the right common carotid artery (CCA), internal carotid artery (ICA), and external carotid artery (ECA), a monofilament nylon suture (about 0.24 mm in diameter) with a rounded tip was inserted through the ECA stump into the ICA and gently advanced to the MCA. Then, in order to monitor blood block, the Laser Speckle Imaging system (moorFLPI-2^™^) was used to detect whether the baseline of brain blood flow was >75% reduced (**Figure S2**). Two hours after cerebral ischemia, filament was removed to restore blood flow (reperfusion). All rats had free access to food and water. At 3 h and 24 h after the ischemia, the separated groups of rats were intravenously injected with indicated dose of CBL (dissolved in saline) or saline alone. In total, 120 rats went through tMCAO operation, among which 6 rats were excluded for hemorrhagic transformation, 10 for unsuccessful occlusion and 9 rats died during the operation.

### Measurement of Neurological Performance and Infarct Size

Rat neurological performance was measured by the following behavioral tests with minor modifications, in a blinded manner. After the tMCAO experiment, the neurological impairment of rats were assessed by Longa’s test: 0, normal function; 1, flexion of the torso and contralateral forelimb after lifting the animal by the tail; 2, circling to the contralateral side but normal posture at rest; 3, reclining to the contralateral side at rest; 4, absence of spontaneous motor activity. Sections were then stained with 2% TTC and placed in the dark area at 37°C. After the brain was stained, the infarct area turned white, while the normal brain tissue remained red. The ratio percentages of the infarct areas to the total brain areas were accessed by morphometric analysis Image-pro plus (Media cybernetics, MD, USA).

### Rotarod Test

To assess the motor behavior recovery after tMCAO with or without treatment of CBL, rats were subjected to a rotating rod test. In this experiment, the rats were placed in a rotating rod from 4× rpm to 40× rpm during the training period, and finally each rat was kept for 300 s without dropping the rod. The latency to fall off the rotating rod was recorded three times daily till 14 days after brain ischemia. And the final data were analyzed as the average value.

### Corner Test

The Corner test was carried out to detect the asymmetrical sensorimotor dysfunction of rats after tMCAO. The experimental device consists of two cardboards forming an angle of 30°. When the rats enter it, the bilateral tentacles will be stimulated, and the rats will stand and face the open end of the angle. Normal rats have the same chances of going left side or right one. However, the rats after tMCAO operation usually turn to the side of the brain injury. The numbers of left and right over 10 trials were recorded.

### Modified Neurological Severity Score

Modified neurological severity score is used mainly to determine the improvement of neurological deficit, thus we used this method to assess the neurological impairment of rats after tMCAO with or without the administration of CBL. The modified neurological severity score (mNSS) contains exercise test, tailing test, the placement test, proprioceptive test (deep sensation, pushing the paw against the table edge to stimulate limb muscles), balance beam test, the experiment of reflex loss and abnormal movement. The Maximum point is 18 and the overall points were assessed by the behavior of rats after tMCAO (normal score, 0; maximal deficit score, 18).

### LPS-Induced Neuroinflammatory Model In Mice

As previously reported, LPS can induce the neuroinflammation in mice (Leite et al., 2016). Firstly, mice were randomly separated into five groups (n = 6–9 of successfully treated rats per group). Mice were intraperitoneally injected with 20 mg/kg, 60 mg/kg, or 100 mg/kg CBL dissolved in the saline or saline alone daily in the 3 consecutive days. On the third day, at 3 h after the administration of CBL or saline in mice, LPS (0.33 mg/kg) dissolved in the saline was intraperitoneally injected according to the body weight of the mice. After 3 h, all the mice were subjected to Open field test to analyze the sickness behavior induced by the LPS. After the behavior test, the blood of the mouse was immediately taken for measurement of pro-inflammatory factors, and the whole cerebral cortex of the mouse was extracted for assessment of inflammatory mediators and related protein expressions.

### Open Field Test

To determine the locomotor activity, the mice after the intraperitoneal administration of 0.33 mg/kg LPS were put into the square open field box (50 × 50 cm). And the total distance traveled, the total time spent and other useful information in the inner zones were recorded by the overhead camera and the final results were analyzed by TopScan software (Anymaze^™^, Stoelting Co). After experiment, the inner and bottom surfaces of the square box must be cleaned to avoid interference information. And the inner zones need to be consistently bright to avoiding interfering with the locomotor activity of mouse. Each mouse was shortly given an overall score for total locomotor activity, including total distance removed, central distance removed, central time spent, time moving towards central area, line crossings, time in the central area, and number exit the central area.

### Measurement of TNF-α by ELISA

The TNF-α ELISA kit was bought from the Becton Dickinson Company (USA). Detection of TNF-α protein was carried out according to the manufacturer’s protocol for the quantification.

### Isolation and Culture of Primary Mouse Microglia

Fifteen newborn mice were disinfected with 75% ethanol, then the brains were put into ice-cold D-HBSS solution, and the vascular membranes were completely removed from it. After 20 min of trypsin digestion, DF_12_ with 10% FBS was added to stop the digestion. Then, the mixture was centrifuged and the precipitation was added to 75 cm^2^ flask coated with poly-L-lysine (PLL, Sigma-Aldrich), and incubated at 37°C and 5% CO_2_. After 14 days of culture, primary mouse microglia were obtained and validated by immunostaining.

### Western Blot Analysis

When finishing the cell experiments, 100 μl of Radio Immunoprecipitation Assay (RIPA) lysis buffer was added after washing cells with phosphate buffered solution (PBS). Cell protein samples were then collected as described previously (Gao et al., 2015) and separated by sodium salt-Polyacrylamide gel electrophoresis (SDS-PAGE) and then transferred to Poly Vinylidene Fluorid (PVDF) membranes. Hereafter, the membranes were blocked with 5% bovine serum albumin for 2 h and then incubated with rabbit antibody of anti-phospho-CREB, rabbit anti-PGC-1α, and rabbit anti-β-actin for 12 h. After washing with Tris buffer saline plus Tween (TBST), the membranes were incubated with the anti-rabbit IgG (1:10,000; Sunshine Bio, China) at room temperature for 1 h. The final bands were measured through chemiluminescence with Bio-Rad ChemiDoc XRS (Bio-Rad, Hercules, California, USA) and protein expression levels were quantified using densitometric analysis and normalized to the levels of β-actin protein.

### RNA Isolation and Quantitative Real-Time PCR

In order to detect the level of expression of the target genes in cells and brain tissues, TRIZOL reagent was added to extract total RNA. Afterwards, according to standard protocol, isolated RNA was reverse-transcribed into cDNA using cDNA synthesis kit. Quantitative PCR (qPCR) was performed at 95°C for 10 min and 40 cycles of 95°C for 15 s, 60°C for 60 s with synthetic primers and SYBR Green. The final results were all normalized as fold change of the target gene/18s *r*RNA. The primers for qPCR are listed in **Table 1**.

**Table 1:** Primers for RT-PCR.

RT-PCR primers	Forward sequence 5’-3’	Reverse sequence 5’-3’
TNF-α (mouse)	GCTGAGCTCAAACCCTGGTA	CGGACTCCGCAAAGTCTAAG
IL-1β (mouse)	TGTGAAATGCCATTTGA	GGTCAAAGGTTTGGAAGCAG
IL-6 (mouse)	CCAGTTGCCTTCTTGGGACTG	CAGGTCTGTTGGGAGTGGTATCC
iNOS (mouse)	CCCAGAGTTCCAGCTTCTGG	CCAAGCCCCTCACCATTATCT
COX-2 (mouse)	TGGGGTGATGAGCAACTATT	AAGGAGCTCTGGGTCAAACT
CD206 (mouse)	CTTCGGGCCTTTGGAATAAT	TAGAAGAGCCCTTGGGTTGA
Arg 1 (mouse)	CTGGTCGGTTTGATGCTA	TGCTTAG CTCTGTCTGCTTTGC
IL-10 (mouse)	GCTCTTACTGACTGGCATGAG	CGCAGCTCTAGGAGCATGTG
TNF-α (rat)	TTCCCAAATGGGCTCCCTCT	GTGGGCTACGGGCTTGTCAC
IL-1β (rat)	TCCAGGATGAGGACCCAAGC	TCGTCATCATCCCACGAGTCA
iNOS (rat)	AGGCCACCTCGGATATCTCT	GCTTGTCTCTGGGTCCTCTG
CD206 (rat)	GGTTCCGGTTTGTGGAGCAG	TCCGTTTGCATTGCCCAGTA
YM 1/2 (rat)	CGGCAGACATTCATCAAATC	GCACCAGGACACTGAAGAGA
Arginase 1 (rat)	TGGACTGGACCCAGTATTCA	CCCAAGAGTTGGGTTCACTT
18s rRNA	CTTTGGTCGTCGCTCCTC	CTGACCGGTTGGTTTTGAT

### Measurement of Lactate Dehydrogenase (LDH) Activity

Primary microglia were cultured at a density of 70–80% in 96-well plates and then treated with different concentrations of CBL for 24 h. Cell supernatants were then collected and assessed by using the Lactate dehydrogenase (LDH) kit and the absorbance was read at 570 nm according to the manufacturer’s instructions.

### Statistical Analysis

The statistical analysis of data was processed using GraphPad Prism Software 7 (La Jolla, CA) and all results are presented as mean ± SEM. Statistical analysis about the frequencies (Longa test and corner test) was put into effect using non-parametric Mann-Whitney test, and the distinction of multiple groups were executed through the One-way ANOVA consistent with Bonferroni’s test or the Two-way ANOVA followed by Bonferroni’s test. The differentiation was recognized significant if the *P* < 0.05 (**Table S1**).

## Results

### CBL Reduced Cerebral Infarction Size, Promoted Neurobehavioral Recovery and Altered the Expression of Inflammatory Mediators in Rats Subjected to tMCAO

**Figure 1:**
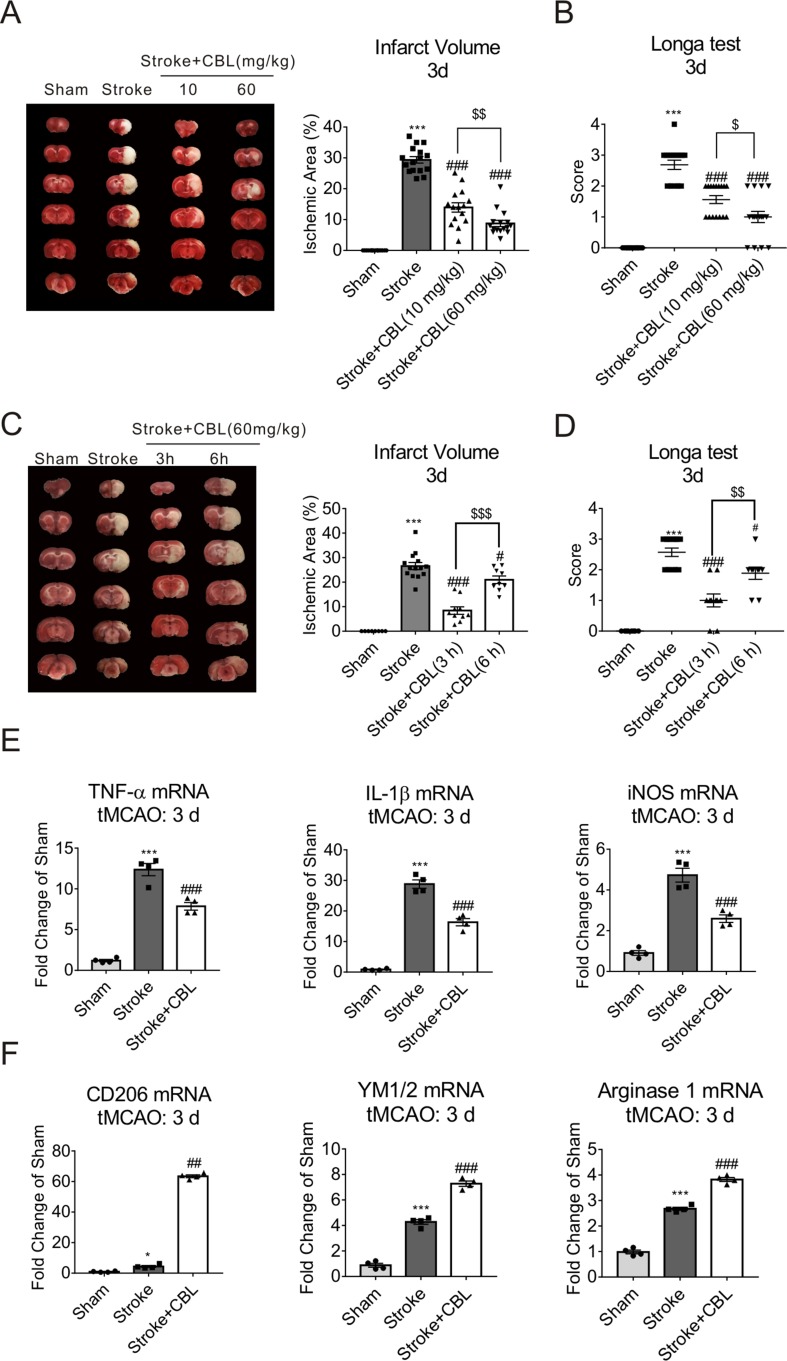
Cerebrolysin (CBL) reduced cerebral infarct volume, promoted sensorimotor recovery, and exhibited anti-inflammatory effect. Rats were intravenously injected with 10 mg/kg or 60 mg/kg CBL at 3 h and 24 h after ischemia, and brains were removed to test histological analysis at 72 h after ischemic stroke. CBL administration at 3 h or 6 h post ischemia, significantly reduced cerebral infarct volume and improved neurologic deficit **(A–D)**. N = 9–15, results are expressed as means ± SEM. ***P < 0.001 versus the Sham group; ^#^*P* < 0.05, ^###^*P* < 0.001, ^$$^*P* < 0.01, ^$ $ $^*P* < 0.001 versus the Stroke group. Post-stroke CBL treatment at 60 mg/kg significantly suppressed the gene expression of pro-inflammatory factors (TNF-α, IL-1β, and iNOS), while promoted the gene expression of anti-inflammatory factors (CD206, YM1/2, Arginase 1) in the ischemic cortex of rats **(E–F)**. N = 4, results are expressed as means ± SEM. ***P < 0.001 versus the Sham group; *P < 0.05, ^##^*P* < 0.01, ^###^*P* < 0.001 versus the Stroke group.

As previously reported, CBL has been used in treating acute ischemic stroke (Zhang et al., 2010; Heiss et al., 2012). Thereby, in order to make sure whether the treatment with CBL can display neuroprotective effects in rats after tMCAO operation, the percentage of the infarct size and neurobehavioral score were analyzed by TTC staining and Longa test, respectively. The stroke group displayed a significant infarct size and neurological deficit. In comparison to stroke group, the infarct area and the neurological deficit of ischemic rats treated with CBL were significantly reduced in a dose-dependent manner (**Figures 1A, B**) and in a time-dependent manner (**Figures 1C, D**). It’s well established that neuroinflammation exerted a crucial role in pathologic process of acute ischemic stroke. Thus, we performed the experiment and found that the mRNA expression of the pro-inflammatory factors (TNF-α, IL-1β, and iNOS) in ipsilateral cerebral cortex was significantly decreased and the mRNA expression of the anti-inflammatory factors (CD206, YM1/2, and Arginase 1) was increased after CBL treatment at 60 mg/kg than the stroke group (**Figures 1E, F**).

### CBL Promoted Long-Term Functional Recovery in Rats Subjected to tMCAO

**Figure 2:**
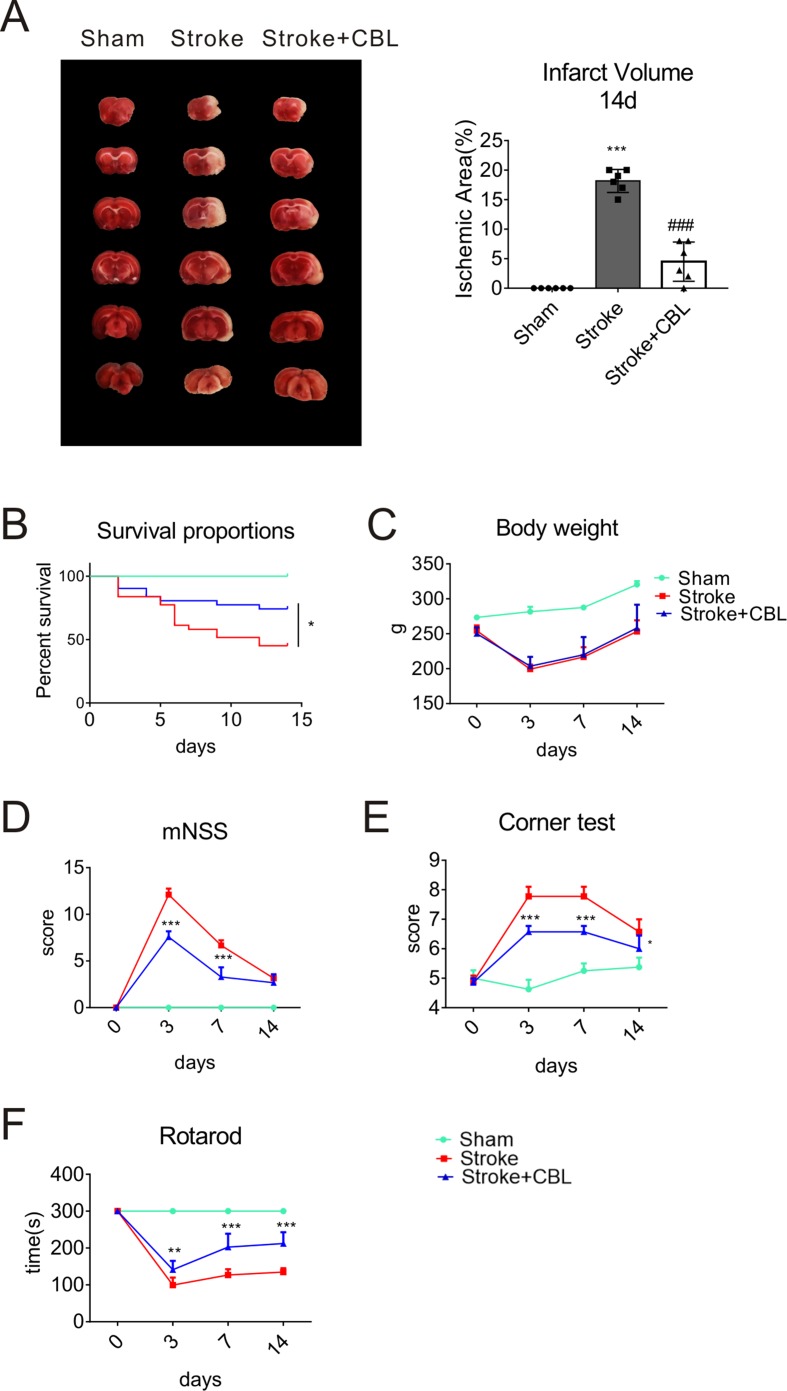
CBL promoted long-term functional recovery in rats subjected to tMCAO. CBL administration significantly reduced the infarct volume at 14 d after ischemia in rats subjected to tMCAO **(A)**. ^###^*P* < 0.001 versus the Stroke group. CBL had no effect on body weight, but significantly increased the survival proportions in rats subjected to tMCAO **(B, C)**. CBL significantly improved the mNSS score in rats after tMCAO operation **(D)**, reduced the numbers of turning to the right **(E)**, and increased the time on the rotating rod **(F)**. Rats were intravenously injected with 60 mg/kg CBL at 3 h and 24 h after tMCAO. N = 8–15, results are expressed as means ± SEM. *P < 0.05, **P < 0.01, ***P < 0.001 versus the Stroke group.

We found that even at 14 day after stroke, CBL also significantly reduced the infarct volume of rats subjected to ischemia (**Figure 2A**). To further test the long-term functional recovery after CBL administration in tMCAO rat model, rats were fulfilled with series of experiments of mNSS, rotarod test, corner test, survival proportions, and body weight from the first day to the fourteenth days after tMCAO. Administration of CBL at 60 mg/kg post-ischemia significantly increased the survival proportions in rats after ischemia, but had no effect on weight loss (**Figures 2B, C**). The stroke group displayed obvious neurological deficits according to the experiments of mNSS, rotarod test, and corner test, which were ameliorated by the administration of CBL (**Figures 2D–F**).

### CBL Ameliorated Depressive Behavior and Reduced Pro-Inflammatory Factors in LPS-Induced Neuroinflammatory Mice

**Figure 3:**
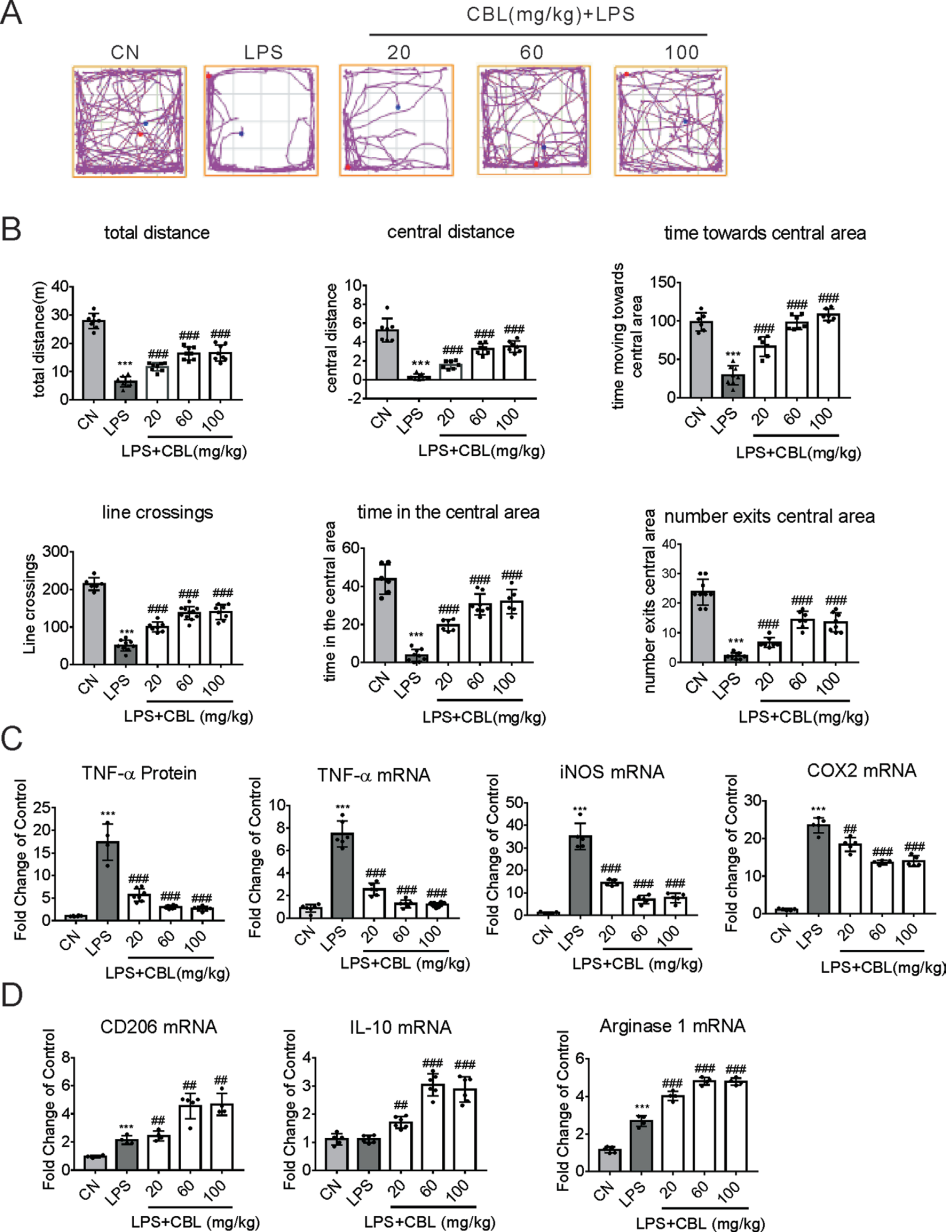
CBL suppressed brain inflammation in neuroinflammatory mice model induced by LPS. **(A)** The typical path of control group (CN, intraperitoneal injection with saline), LPS group (intraperitoneal injection with 0.33 mg/kg LPS), LPS+CBL group (intraperitoneal injected with 0.33 mg/kg LPS plus 20 mg/kg, 60 mg/kg or 100 mg/kg CBL). **(B)** CBL increased total distance, central distance, time towards the central area, line crossings, time in the central area, number exits the central area in a concentration-dependent manner in mice subjected to LPS stimulation compared with the LPS group. N = 6–10, results are expressed as means ± SEM. **(C, D)** CBL reduced serum TNF-α level, decreased the gene expression of pro-inflammatory mediators (TNF-α, COX2, and iNOS), and promoted the gene expression of anti-inflammatory mediators (CD206, IL-10, and Arginase 1) in the cerebral cortex of mice subjected to LPS treatment in a dose-dependent manner. N = 4–8, results are expressed as means ± SEM. ***P < 0.001 versus the CN group; ^##^*P* < 0.01, ^###^*P* < 0.001 versus the LPS group.

As previously demonstrated, in tMCAO model, neuroinflammation exerts considerable influence (Leite et al., 2016). Therefore, we selected the model of LPS-induced neuroinflammation to verify again whether CBL possesses the anti-inflammatory effects (Zhao et al., 2017). In this model, all mice survived and displayed no abnormal behavior. In the Open field test, the group injected with LPS showed clear sickness behavior, which was ameliorated by the administration of CBL in a dose-dependent manner (**Figure 3A**).More specifically, treatment with CBL significantly increased the LPS-induced decrease in total distance, central distance, central time, time moving towards central area, line crossings, time in the central area, and number exit the central area (**Figure 3B**).

Furthermore, LPS administration promoted the protein expression of TNF-α in the serum of mice, which was reduced by CBL treatment in a dose-dependent manner (**Figure 3C**). Subsequently, we extracted the cerebral cortex of the mouse and found the gene expression of pro-inflammatory mediators has been largely elevated in the group of LPS injection (TNF-α, COX2, and iNOS). Compared with it, CBL administration evidently ameliorated this phenomenon (**Figure 3C**). Correspondingly, CBL significantly increased anti-inflammatory mediator gene expression (CD206, Arginase 1, and IL-10) (**Figure 3D**). These data demonstrated CBL could promote microglia activation towards the anti-inflammatory phenotype in the LPS-induced neuroinflammatory model.

### CBL Promoted Microglial Transition Toward Anti-Inflammatory Phenotype in Primary Mouse Microglia Against LPS-Induced Inflammatory Response

**Figure 4:**
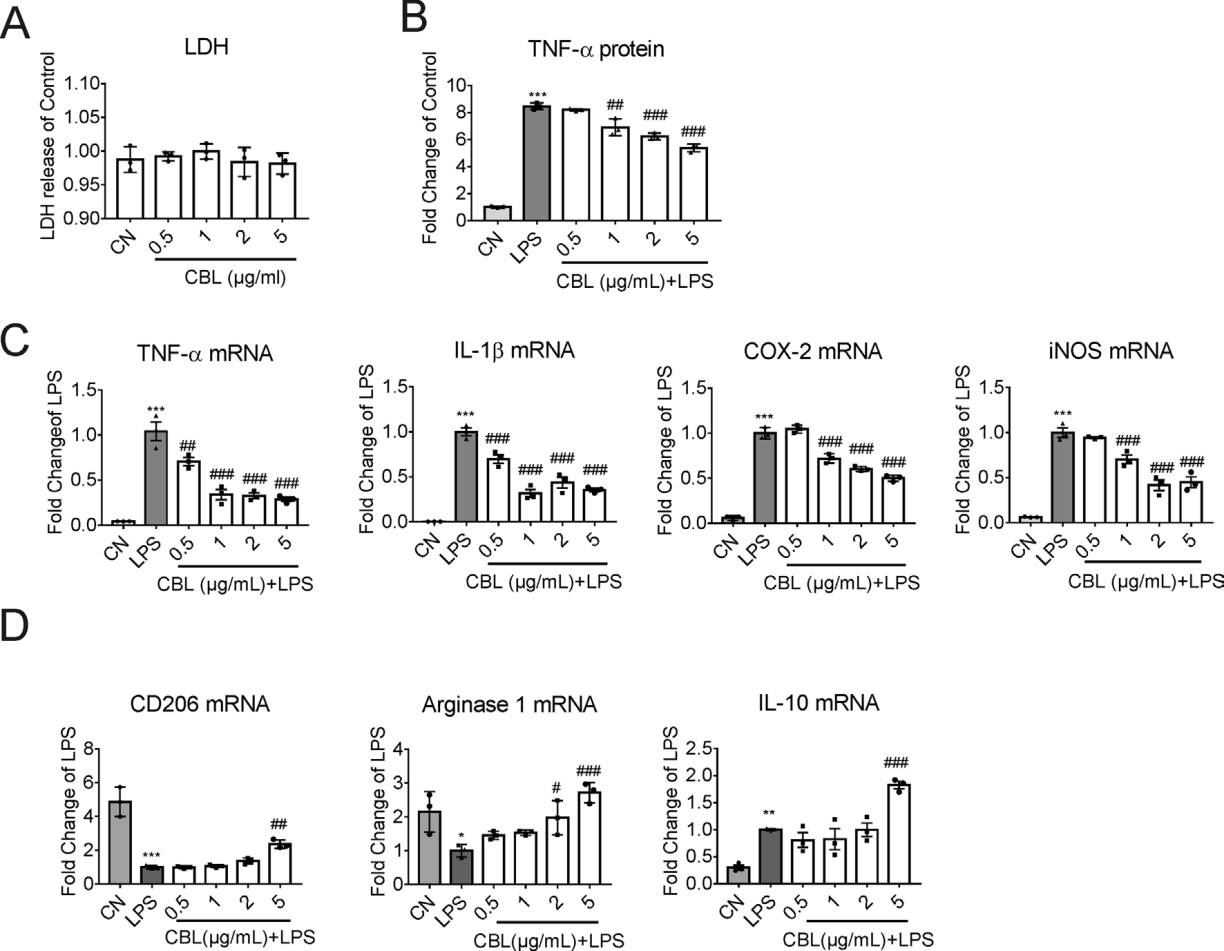
CBL suppressed LPS-induced inflammatory response in primary mouse microglial cells. **(A)** Cells were treated with different concentrations of CBL for 24 h to assess the cytotoxicity by LDH kit. **(B)** CBL could decrease the release of TNF-α in microglial cells after LPS incubation as assessed by ELISA kit. Primary microglial cells were pre-incubated for 3 h with different concentrations of CBL followed by 100 ng/mL LPS incubation for another 2 h. **(C, D)** CBL reduced the gene expression of pro-inflammatory factors (TNF-α, IL-1β, COX-2, and iNOS) and promoted the gene expression of anti-inflammatory factors (CD206, Arginase 1, and IL-10) as assessed by quantitative RT-PCR. Primary microglial cells were incubated with different concentrations of CBL for 3 h followed by 100 ng/mL LPS incubation for another 2 h to determine the indicated genes expression. Results are shown as means ± SEM (n = 3). *P < 0.05, **P < 0.01, ***P < 0.001 versus the control (CN) group (Saline); ^#^*P* < 0.05, ^##^*P* < 0.01, ^###^*P* < 0.001 versus the LPS group.

To ascertain the anti-inflammatory effects of CBLin *in vitro* model, we stimulated primary mouse microglia with 100 ng/ml LPS. When the concentration arrange of CBL was from 0.5 to 5 μg/ml, it displayed no toxicity in primary microglia (**Figure 4A**). In order to further determine the optimal anti-inflammatory dose of CBL, we collected the cell culture medium of primary microglia after LPS stimulation and found that 5 μg/ml is the best concentration to inhibit inflammation (**Figure 4B**). Meanwhile, 5 μg/ml of CBL significantly decreased LPS-induced elevation of pro-inflammatory factor gene expression (TNF-α, IL-1β, iNOS, and COX-2) (**Figure 4C**), and increased anti-inflammatory mediator gene expression (**Figure 4D**). The above results implied that CBL promoted the switch of microglia towards an anti-inflammatory phenotype in primary microglia cultures.

### CBL Reduced Expressions of Phosphorylated P38 and JNK After LPS Stimulus and Its Anti-Inflammatory Effect Was Mediated Partly Through the CREB/PGC-1α Pathway in Primary Microglia

**Figure 5:**
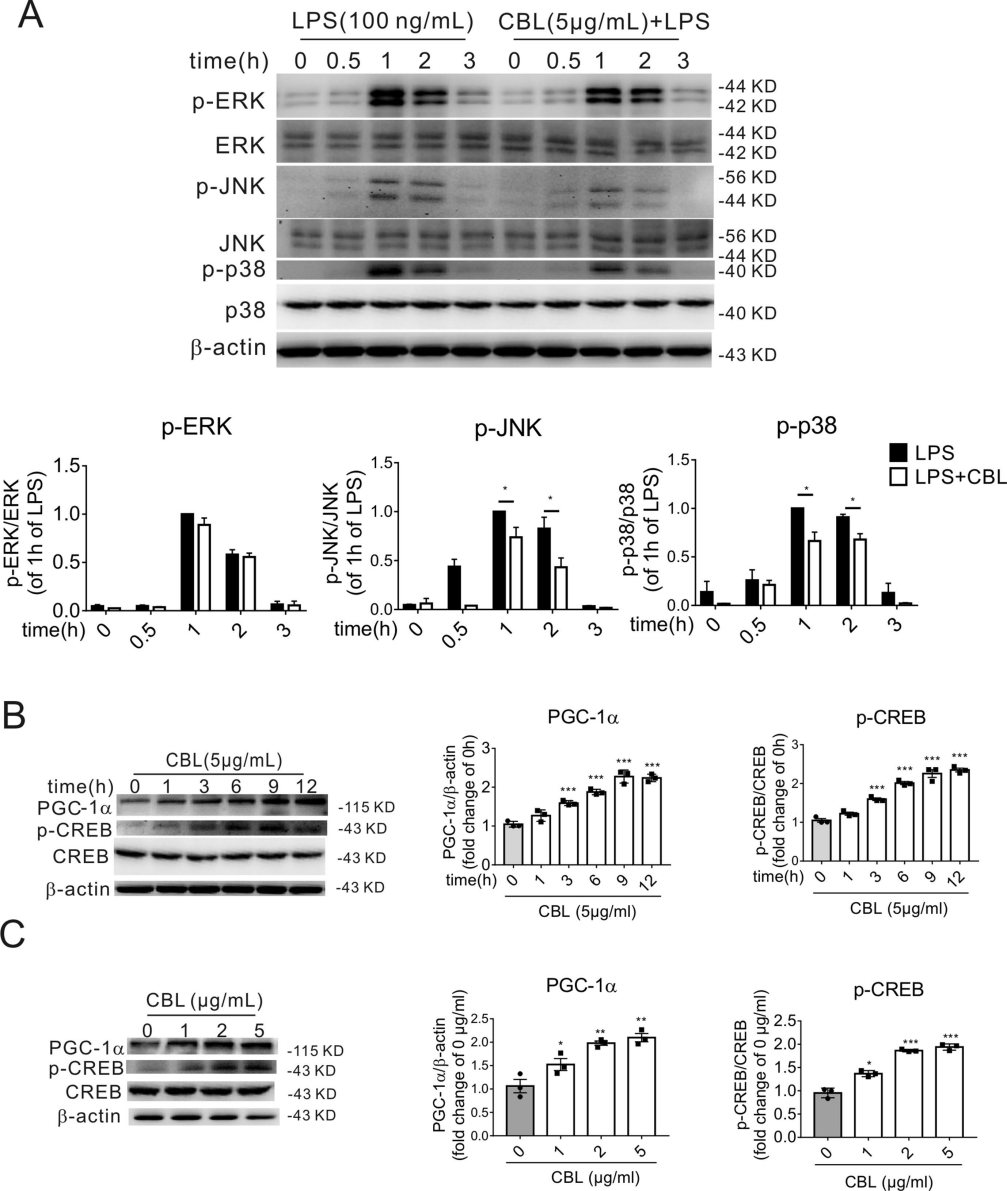
MAPK pathway and CREB/PGC-1α pathway are involved in the CBL inhibition of inflammatory response in primary mouse microglial cells. **(A)** CBL significantly reduced the protein expression of phosphorylated JNK and p38 MAPK in LPS-stimulated primary mouse microglial cells. Cells were pre-incubated with 5 μg/mL CBL for 3 h followed by 100 ng/ml LPS incubation for indicated different times to assess the protein expression. Results are expressed as means ± SEM (n = 3). *P < 0.05 versus the LPS group. **(B, C)** CBL increased the protein expression of phosphorylated CREB (p-CREB) and PGC-1α in the time- and dose-dependent manners in primary mouse microglial cells. Cells were treated with 5 μg/mL CBL for different times or different concentrations of CBL. Data are shown as the mean ± SEM (n = 3) after normalization to the control. **P< 0.01, ***P < 0.001 versus control (0 h or 0 μg/ml).

LPS activated the phosphorylation of c-Jun N-terminal kinase (JNK), extracellular signal-regulated kinase 1/2 (ERK1/2) and p38 mitogen-activated protein kinase (p38 MAPK) in primary microglia in a time-dependent manner, and 1 h was the highest point after stimulation with LPS. In primary mouse microglia, CBL significantly inhibited LPS-induced phosphorylation of p38 MAPK and JNK, but displayed weakly inhibitory effect on ERK1/2 phosphorylation (**Figure 5A**).

As previously reported, MAP kinases can activate phosphorylation of CREB (p-CREB), while CREB is a transcription factor that regulates the expression of PGC-1α (Raefsky and Mattson, 2017). Therefore, we examined the effect of CBL on the protein expression of p-CREB and PGC-1α. The administration of CBL enhanced the expression of PGC-1α and p-CREB in primary microglia cells in the time-dependent manner (**Figure 5B**) and dose-dependent manner (**Figure 5C**). The maximum effective concentration of CBL was 5 μg/mL and the expression levels of related protein reached maximum at 9 h after exposure to CBL.

### The Specific CREB Inhibitor 666-15 Attenuated the Anti-Inflammatory Effects of CBL in LPS-Stimulated Microglia and Anti-Ischemic Effect of CBL in Rats

**Figure 6:**
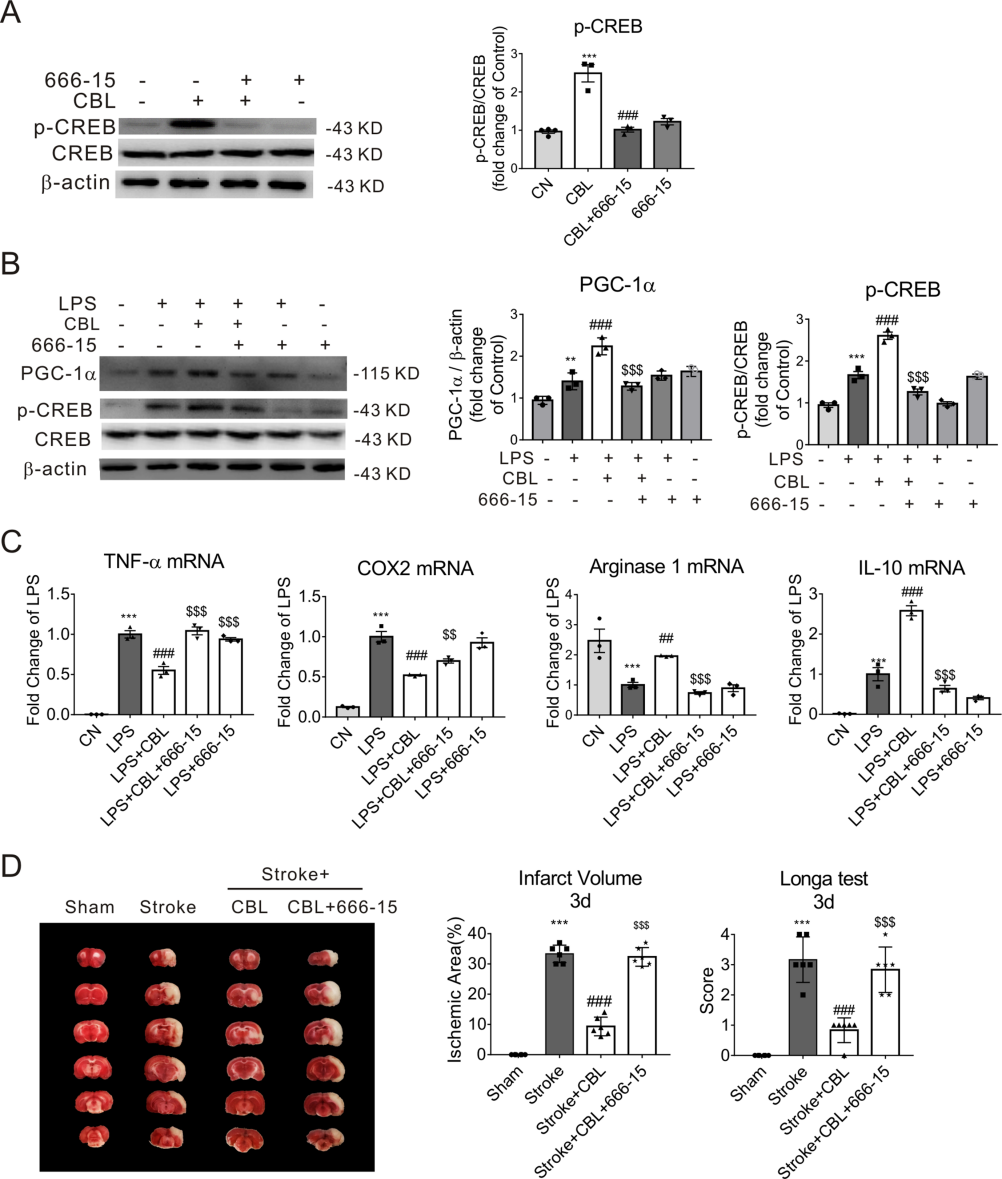
The anti-inflammatory effect of CBL was abolished by a specific CREB inhibitor 666-15 compound in the primary mouse microglial cells, and 666-15 also attenuated the anti-ischemic effect of CBL. **(A)** 666-15 reduced protein expression of phosphorylated CREB (p-CREB) in primary mouse microlgia. Cells pre-incubated with 666-15 compound (1 μM) for 12 h were treated with 5 μg/mL CBL for another 3 h. **(B)** 666-15 compound attenuated the CBL enhancement of the protein expression of PGC-1α and p-CREB in primary microglia. Cells pre-incubated with 666-15 compound (1 μM) for 12 h were treated with 5 μg/mL CBL for another 3 h, and then added 100 ng/ml LPS for 2 h. **(C)** 666-15 compound reversed the CBL inhibition of pro-inflammatory factors gene expression and the promotion of anti-inflammatory factors gene expression. Results are shown as means ± SEM (n = 3). **P < 0.01, ***P < 0.001 versus the control group (CN); ^##^*P* < 0.01, ^###^ *P* < 0.001 versus the CBL group or LPS group; ^$$^*P* < 0.01, ^$$$^*P* < 0.001 versus the LPS+CBL group. **(D)** The CREB inhibitor 666-15 compound reversed the anti-ischemic effect of CBL in rats subjected to tMCAO. Rats were first intraperitoneally injected with 10 mg/kg 666-15 compound for 30 min, followed by intravenous injection with 60 mg/kg CBL at 3 h and 24 h after ischemia, and brains were removed to test histological analysis at 72 h after ischemic stroke. N = 6, results are expressed as means ± SEM. ***P < 0.001 versus the Sham group; ^###^*P* < 0.001 versus the Stroke group; ^$$$^*P* < 0.001 versus the Stroke+CBL group.

The compound 666-15 is a potent and selective CREB inhibitor which inhibits transcription activity of CREB. When primary microglia were treated with 666-15 for 12 h, the level of p-CREB protein was markedly decreased (**Figure 6A**). When stimulated with LPS, 666-15 also inhibited the expression of p-CREB and PGC-1α after the administration of CBL in LPS-stimulated primary mouse microglia (**Figure 6B**). The inhibition of CREB also reversed the effect of CBL on reducing pro-inflammatory factor gene expression (TNF-α, COX2) (**Figure 6C**) and increasing anti-inflammatory mediator gene expression (Arginase 1 and IL-10) (**Figure 6C**). These data indicate that the CREB-mediated PGC-1α activation may be involved in the anti-inflammatory effects of CBL in primary microglia.

In addition, we performed animal experiment to determine whether CREB inhibitor could prevent the anti-ischemic effect of CBL. As shown in **Figure 6D**, we found that the CREB inhibitor 666-15 compound could significantly reverse the beneficial effect of CBL in rats subjected to tMCAO, which further confirms that the CREB pathway is involved in the anti-ischemic effect of CBL.

### CBL Activated the CREB/PGC-1α Pathway in the Brain Cortex of LPS-Induced Neuroinflammatory Mice or Rats Subjected to tMCAO

**Figure 7:**
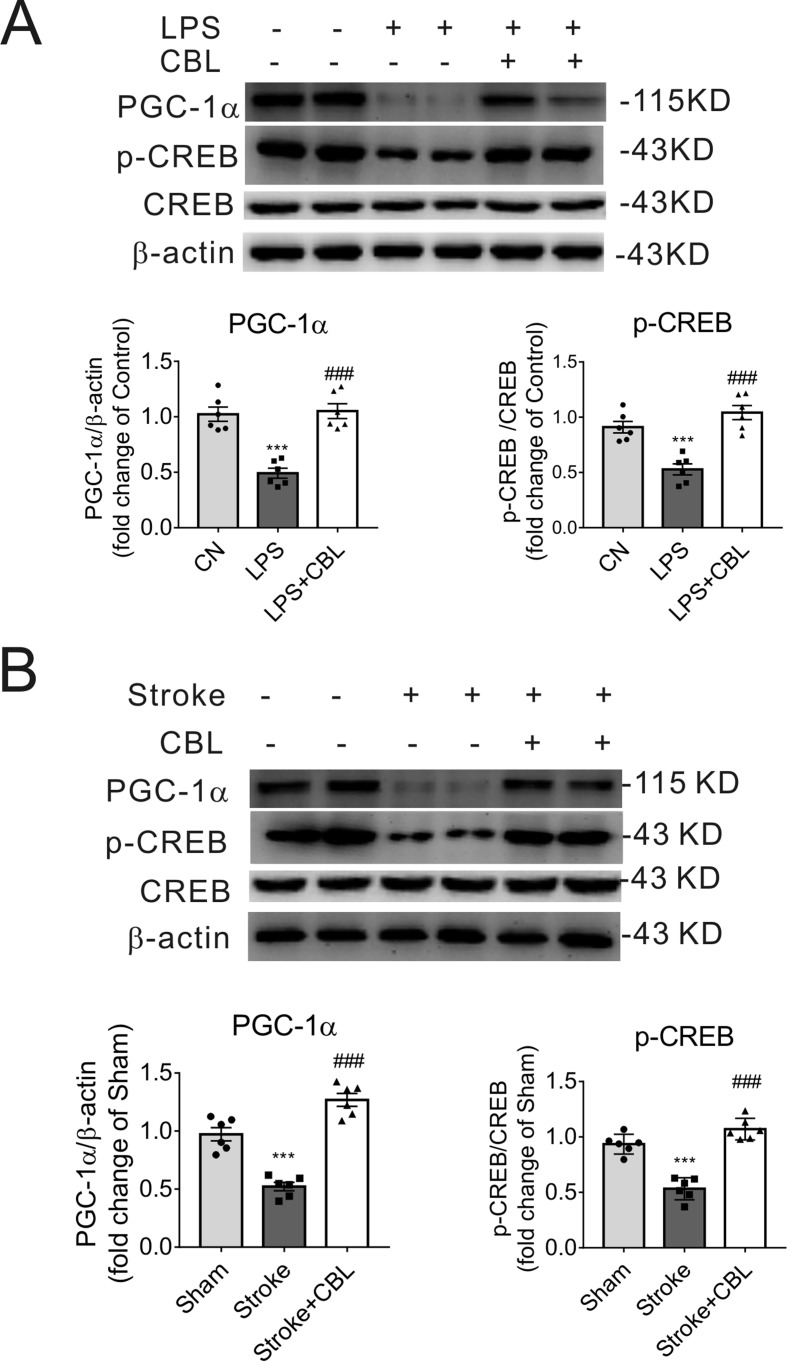
CBL enhanced CREB activity and PGC-1α protein expression in LPS-induced neuroinflammatory mice model and cerebral ischemic rat model. CBL (60 mg/kg) administration significantly increased the protein expressions of PGC-1α and phosphorylated CREB (p-CREB) in the cerebral cortex of neuroinflammatory mice subjected to LPS injection **(A)** or in the ischemic cortex of the brain of rats subjected to tMCAO at 3 d after ischemia **(B)**. Results are shown as means ± SEM (n = 6). ***P < 0.001 versus the Control group (CN) or Sham group; ^###^*P* < 0.001 versus the LPS group or the Stroke group.

In LPS-induced neuroinflammation model, the group administrated with CBL significantly enhanced the protein expression of PGC-1α and CREB phosphorylation (p-CREB) compared with LPS group (**Figure 7A**). Accordingly, in the ipsilateral brain cortex of rats after tMCAO, treatment with CBL markedly increased the protein expression of PGC-1α and CREB phosphorylation in comparison with the stroke group (**Figure 7B**).

In conclusion, the above results indicate that the administration of CBL exerted anti-inflammatory effects and ameliorated cerebral ischemic stroke injury through activation of the CREB/PGC-1α signaling pathway.

## Discussion

Neuroinflammation plays an important role in ischemic stroke, in which the pro-inflammatory phenotype of microglia release pro-inflammatory mediators, producing neurotoxicity, and anti-inflammatory phenotype of microglia release anti-inflammatory mediators to promote neurogenesis and functional recovery (Voet et al., 2018). As CBL has been applied for the clinical treatment of ischemic stroke, our study, therefore, was designed to test whether CBL has anti-inflammatory effects to show beneficial role in cerebral ischemic stroke. Our main experimental findings are: 1) In tMCAO rats, CBL reduced ischemic infarct volume and promoted neurobehavioral recovery by reducing pro-inflammatory factors expression and increasing anti-inflammatory factors expression. 2) LPS-stimulated inflammatory model in primary microglia and LPS-induced neuroinflammatory mice model demonstrated that CBL promoted microglia polarization into anti-inflammatory type *in vitro* and *in vivo*. 3) The CREB/PGC-1α pathway was found to play an important role in CBL effects.

CBL has been widely used for the treatment of brain-related diseases as porcine brain-derived peptides preparation administered parenterally (Ziganshina et al., 2017). As previously reported, the administration of CBL could help treat acute ischemic stroke clinically as a neuroprotective drug by promoting neuronal survival, neuroprotection, neuroplasticity, neurogenesis, ameliorating spatial learning and memory deficits and improving passive avoidance behavior (Zhang et al., 2010; Heiss et al., 2012). In addition, CBL has been reported to treat Alzheimer’s disease and vascular dementia (Plosker and Gauthier, 2009). The previous study by Barakat et al. (2014) reported that CBL pretreatment before ischemia prevented ischemic brain injury by reduced leukocyte infiltration and proinflammatory factors expression. However, in this study, for the first time, we found that CBL administration post-ischemia significantly ameliorated ischemic brain injury and improved the long-term neurological functional recovery through attenuation of neuroinflammation. Further mechanistic studies show that CBL prevented microglial M1 polarization and promoted microglial M2 polarization mediated by CREB/PGC-1α pathway. Our findings may provide the clinic relevant evidence for the beneficial effects of CBL on the treatment of stroke patients.

Microglia, the resident macrophages in the brain, are the first step of defense against the damage of central nervous system (Chen and Trapp, 2016; Kluge et al., 2017; Hersh and Yang, 2018; Xu et al., 2018). Once ischemic brain injury occurs, microglia rapidly migrate to the lesion area (Cherry et al., 2014). The pro-inflammatory phenotype, also designated as a classically activated microglia, can display enhanced expression of a series of pro-inflammatory cytokines (IL-1β, IL-6, TNF-α, CCL2, and CXCL10), reactive oxygen species (ROS), inducible nitric oxide synthase (iNOS), and nitric oxide (NO), exacerbating tissue damage. However, on the other hand, the anti-inflammatory phenotype, also called an alternately activated microglia, is capable of removing debris and producing anti-inflammatory cytokines (Arginase 1, YM 1/2, IGF-1, CD206, and IL -10) and trophic factors such as insulin-like growth factor 1 (IGF-1), brain source-derived neurotrophic factor (BDNF) (Coull et al., 2005), promoting tissue repair and remodeling (Kluge et al., 2017). In addition, classically activated microglia cells can be detected with obvious increase in target signaling pathways, including NF-κB, the phosphorylation of p38, JAK/STAT, JNK, and ERK1/2 during neuroinflammation (Kaminska et al., 2016). Thus, limiting the expression of these pathways will help to control neuroinflammation (Goldmann et al., 2013). In the *in vivo* and *in vitro* neuroflammation models, CBL administration promoted the transformation of microglia from pro-inflammatory polarization to anti-inflammatory polarization, and decreased the protein expression of phosphorylated p38 MAPK and JNK in mouse primary microglial cells.

CREB exerts an important role in neuroprotection (Paramanik and Thakur, 2013). CREB is phosphorylated at serine 133 site, which in turn binds to DNA sequences and regulates downstream genes (Carlezon et al., 2005). On the one hand, the phosphorylation of CREB is related with the expression of BDNF, protecting brain tissues. On the other hand, this region of serine 133 site is also the area where RelA component of NF-κB interacts with CBP/p300 (Suliman et al., 2010; Wen et al., 2010). Therefore, phosphorylation of CREB competitively inhibits NF-κB. In addition, CREB can bind to the promoter of IL-10 to promote its transcription, exerting an anti-inflammatory effect (Guindi et al., 2018). What’s more, the expression of PGC-1α can be activated by cAMP/PKA or p38 MAPK and the downstream gene of p38 MAPK, while CREB regulates the activation of PGC-1α through a common CRE sequence in the promoter of PGC-1α. CBL can increase the phosphorylation of CREB *in vivo* and *in vitro*. Once the pathway of CREB was blocked by specific CREB inhibitor, the anti-inflammatory effect of CBL was abolished (Saura and Cardinaux, 2017). Specifically, after the tMCAO experiments and LPS-induced brain inflammation, the expression level of phosphorylated CREB in the group with CBL administration was much higher than the stroke group or the LPS-treated group. In this experiment, CBL acted on CREB pathway in mouse primary microglial cells in the time-dependent and dose-dependent manners, which was reversed with the administration of CREB inhibitor 666-15 (Herzig et al., 2001). In general, these results suggested that CBL played anti-inflammatory effects by promoting CREB phosphorylation to increase CREB activity.

PGC-1α, peroxisome proliferator-activated receptor γ coactivator 1α, exerts large influences on neurodegenerative disease through managing mitochondrial biogenesis and limiting oxidative stress (Chen et al., 2011). LPS and TNF-α can inhibit the expression of PGC-1α. Conversely, AMPK and CREB can promote the activation of PGC-1α (Lin et al., 2005). After activation, PGC-1α is capable of binding to the P65 subunit of NF-κB to promote the formation of complexes, thereby inhibiting the releasing of anti-inflammatory mediators and limiting the occurrence of inflammatory reactions (Salminen et al., 2011; Kauppinen et al., 2013). It has been reported that NF-κB is required for the anti-inflammatory phenotype transition (Ghosh and Hayden, 2008), and PGC-1α is also involved in the process under certain conditions. When the expression of PGC-1α is decreased, it promotes the transition towards anti-inflammatory phenotypes of microglia (Yang et al., 2017). In this experiment, we demonstrated that CBL was capable of promoting the expression of PGC-1α in the dose-dependent and time-dependent manners *in vitro*. Additionally, in the tMCAO rat model and LPS-induced neuroinflammatory mice model, the expression of PGC-1α in the group with CBL administration was highly promoted compared to the stroke group. The above observations implied that activation of PGC-1α probably facilitated the switch of pro-inflammatory phenotype of microglia/macrophages towards the anti-inflammatory phenotype.

It has been reported that CBL could significantly increase the expression of phosphorylated CREB (p-CREB) in the hippocampus of rats (Liu et al., 2017). Several reported studies indicate that the expression of PGC-1α may be mediated by the PKA–CREB pathway, and CREB in combination with cAMP could induce the expression of PGC-1α in the liver (Herzig et al., 2001; Louet et al., 2002; Herzig et al., 2003; Yao et al., 2016). Therefore, we speculate that CBL may increase cAMP in the brain to activate PKA, and then promote CREB phosphorylation to increase its transcriptional activity, resulting in the expression of PGC-1α. We will examine this hypothesis in the next study.

In addition, white matter accounts for the vast majority of the human brain. When stroke occurs, the inflammatory response in white matter is more persistent and intense (Peng et al., 2019). Moreover, the treatment of white matter can promote long-term behavioral function and memory function recovery in stroke patients (Qin et al., 2018). Most of the drugs treating stroke that have been proven to have good efficacy in animals cannot be applied to humans, possibly because they lack the ability of promoting the recovery of white matter damage. Whether CBL has the beneficial effects on ameliorating white matter injury caused by neuroinflammation will be conducted in future.

**Figure 8:**
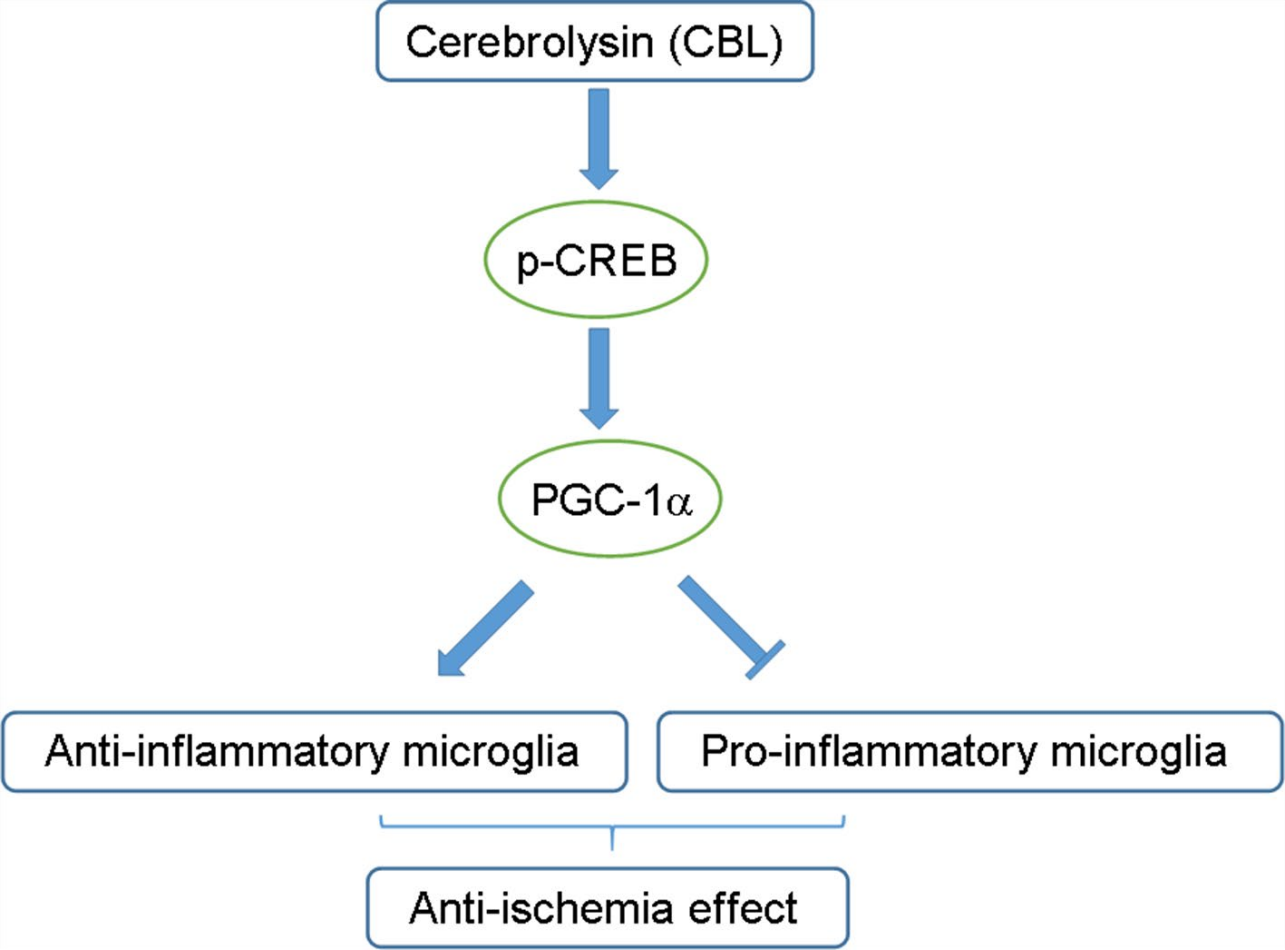
Schematic diagram of the proposed mechanisms for the anti-neuroinflammatory effects of CBL. The CREB/PGC-1α signaling pathway may be partly involved in the anti-neuroinflammatory and anti-ischemic effects of CBL by promoting the pro-inflammatory phenotype of microglia toward anti-inflammatory phenotype.

In conclusion, this study firstly demonstrated that CBL, neuroprotective drug widely used in clinic, was capable of inhibiting neuroinflammation *in vitro* and *in vivo via* the CREB/PGC-1α pathway and ameliorating cerebral ischemic injury in rats subjected to tMCAO (**Figure 8**). These findings may provide additional mechanism responsible for CBL anti-brain ischemic stroke and may also facilitate the discovery of a range of drugs that act on CREB/PGC-1α pathway to be further developed into therapeutic agents for ischemic stroke.

## Data Availability Statement

The datasets generated for this study are available on request to the corresponding author.

## Ethics Statement

All experiments were carried out in accordance with the Guide for the Care and Use of Laboratory Animals of the National Institute of Health. Animals used were approved by the Committee of Experimental Animals in Jiangsu Province and the Ethics Committee of China Pharmaceutical University.

## Author Contributions

XG, YW, and TP developed the conception and design of the study. XG, YW, GK, SZ, TH, YL, and YX performed the experiments and analyzed the data. YW, LZ, and TP revised the manuscript. All authors contributed to the final manuscript and approved it for publication.

## Funding

This study was supported by the National Natural Science Foundation of China (81973512, 81703530, 81773995), the Natural Science Foundation of Jiangsu Province (BK20160032, BK20170859), the Opening Project of Zhejiang Provincial Preponderant and Characteristic Subject of Key University (Traditional Chinese Pharmacology), Zhejiang Chinese Medical University (No. ZYAOX2018001), Double First-Class Project of China Pharmaceutical University (CPU2018GY06, CPU2018GY20), and the Six Talent Peaks Project of Jiangsu Province (TP).

## Conflict of Interest

Authors TH and YL were employed by company of Guangdong Long Fu Pharmaceutical Co., Ltd. The remaining authors declare that the research was conducted in the absence of any commercial or financial relationships that could be construed as a potential conflict of interest.

## Abbreviations

Arg 1, arginase 1; BDNF, brain-derived trophic factor; CNS, central nervous system; CBL, cerebrolysin; COX-2, Cyclooxygenase-2; CREB, cAMP response element binding; CBP, CREB binding protein; CRE, Cyclic AMP Responsive Element; CCA, common carotid artery; ECA, external carotid artery; ERK, extracellular signal-regulated kinase; iNOS, inducible nitric oxide synthase; ICA, internal carotid artery; LPS, lipopolysaccharide; LDH, Lactate dehydrogenase; IL-1β, interleukin-1β; IL-6, interleukin-6; IL-10, interleukin-10; mNSS, Modified neurological severity score; MAPK, mitogen-activated protein kinase; NF-κB, nuclear factor-κ-gene binding; PVD, PolyVinylidene Fluorid; PLL, poly-L-lysine; PBS, phosphate-buffered saline; PGC-1α, peroxisome proliferator-activated receptor gamma co-activator 1α; RT-PCR, Real time-polymerase chain reaction; qPCR, quantitative PCR; rt-PA, recombinant tissue plasminogen activator; ROS, reactive oxygen species; RIPA, Radio Immunoprecipitation Assay; SDS-PAGE, sodium salt polyacrylamide gel electrophoresis; STAT, Signal Transducers and Activators of Transcription; TLR4, Toll-like receptor 4; SD rats, Sprague-Dawley rats; tMCAO, transient middle cerebral artery occlusion; TBST, Tris buffer saline plus tween; TNF-α, Tumor necrosis factor-alpha; TTC, 2,3,5-Triphenyltetrazolium chloride.
