# Supplementary material for: Cerebrolysin Ameliorates Focal Cerebral Ischemia Injury Through Neuroinflammatory Inhibition via CREB/PGC-1α Pathway
Source: Front Pharmacol. 2019 Oct 22;10:1245. doi: 10.3389/fphar.2019.01245 (PMC6818051; doi:10.3389/fphar.2019.01245)
Supplement: Supplementary file 1 [file DataSheet_1.docx]

**Supplementary Material**

**Methods**

**Cortical cerebral blood flow (CBF) measurement**

CBF was measured using the Laser Speckle Imaging system (moorFLPI-2™). All male rats were subjected to repeated measurements of regional cerebral blood flow (rCBF) before and during tMCAO as well as 10 min after reperfusion. CBF changes were expressed as a percentage of pre-MCAO baseline values. Animals that died or failed to display an rCBF reduction to 30% of the baseline using Laser Speckle Imaging system were excluded from further analyses.

**Figures**

**Figure S1.** Experimental design. (A) Experimental design of tMCAO. To determine the effects of cerebrolysin (CBL) on tMCAO and therapeutic window, a total of 120 rats were divided into six groups: Sham, Stroke, Stroke+CBL (10 mg/kg) at 3 h and 24 h after ischemia, Stroke+CBL (60 mg/kg) at 3 h and 24 h after ischemia, Stroke+CBL (60 mg/kg) at 6 h and 24 h after ischemia, Stroke+CBL (60 mg/kg)+666-15 (10 mg/kg) at 3 h and 24 h after ischemia. (B) The long-term functional recovery effect of CBL in rats after tMCAO. A total of 90 rats were divided into three groups: Sham, Stroke group at 3 h and 24 h after ischemia, Stroke+CBL (60 mg/kg) at 3 h and 24 h after ischemia. (C) Experimental design of LPS-induced neuroinflammation mice model. Experiment 3 was to measure the effects of CBL in LPS-induced neuroinflammatory mice model. A total of 80 C57BL/6 mice were divided into five groups: Control group, LPS (0.33 mg/kg), LPS (0.33 mg/kg)+CBL (20 mg/kg), LPS (0.33 mg/kg)+CBL (60 mg/kg), LPS+CBL (100 mg/kg).


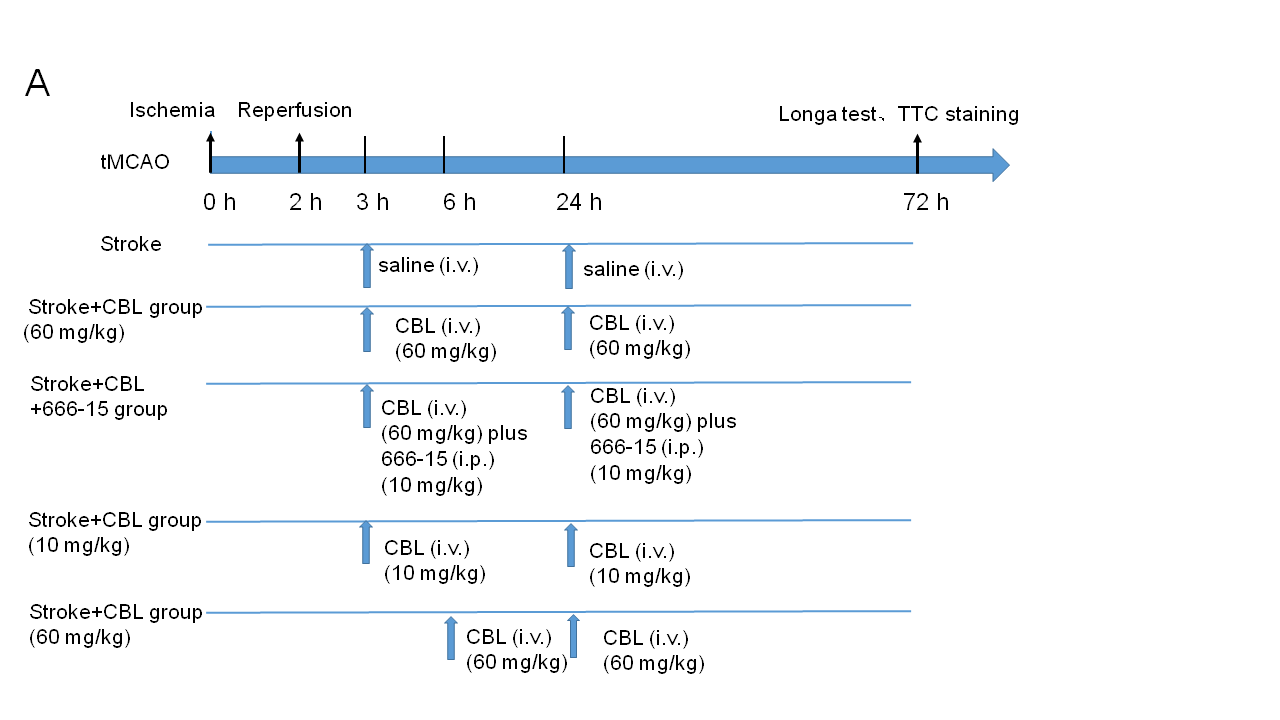


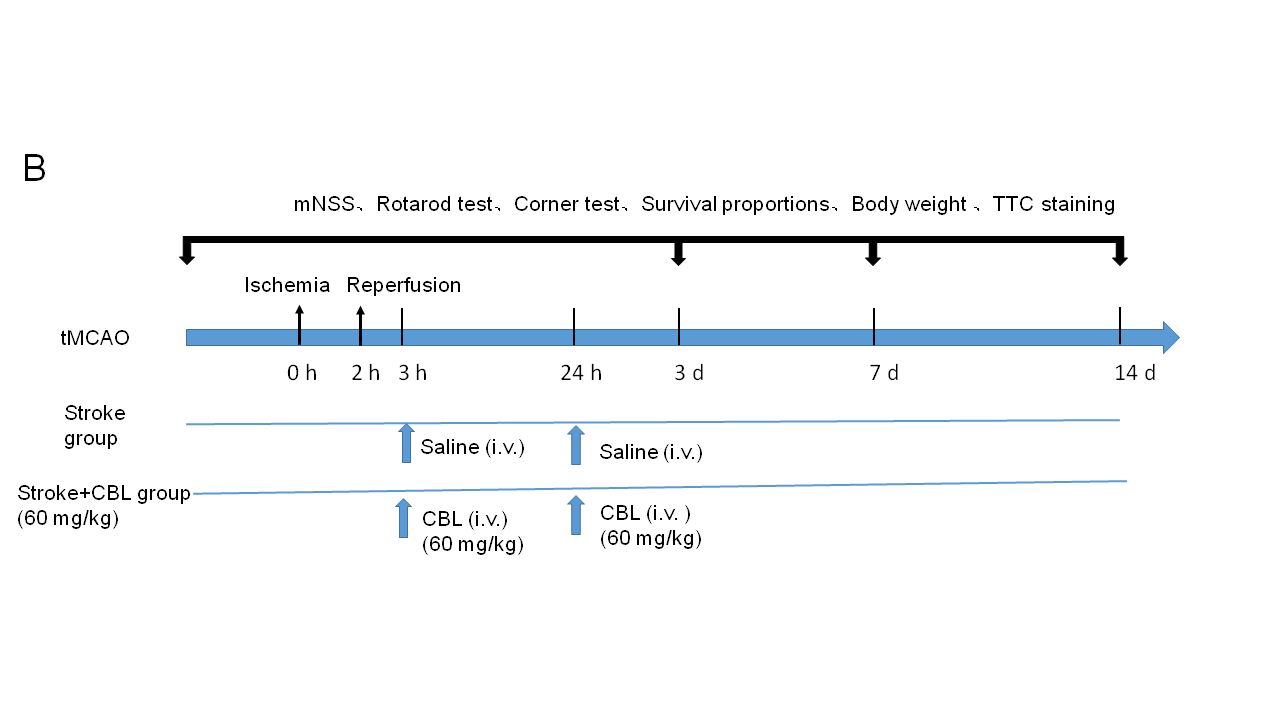


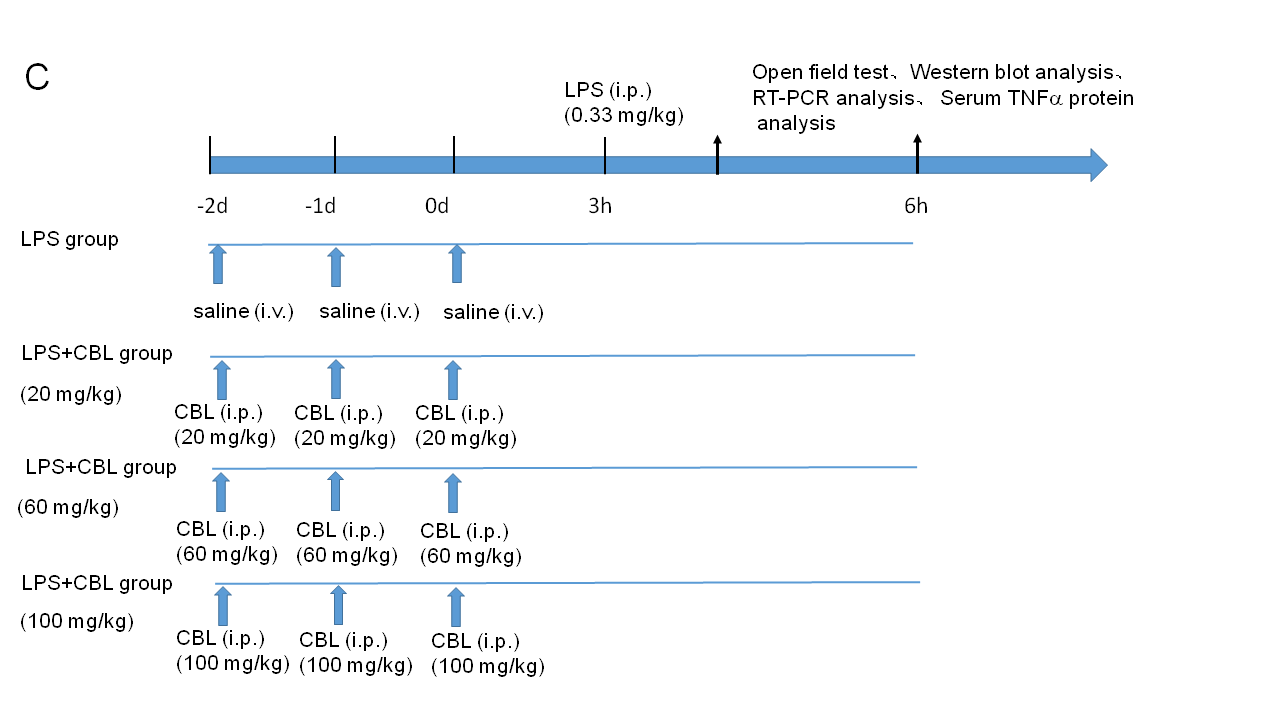


**Figure S2.** Regional cerebral blood flow in the Stroke group and Stroke+CBL group. Regional cerebral blood flow (rCBF) was monitored by the Laser Speckle Imaging system (LSCI) 10 min before and during occlusion, and 10 min after reperfusion. Data were expressed as % of baseline before occlusion. Values shown are mean ± SD. Significant differences was detected between the sham group and stroke group (****P* < 0.001), and between the Occlusion group and Reperfusion group (^###^*P*< 0.001). However, no obvious difference was observed between the Stroke+CBL (60 mg/kg) group and Stroke group.


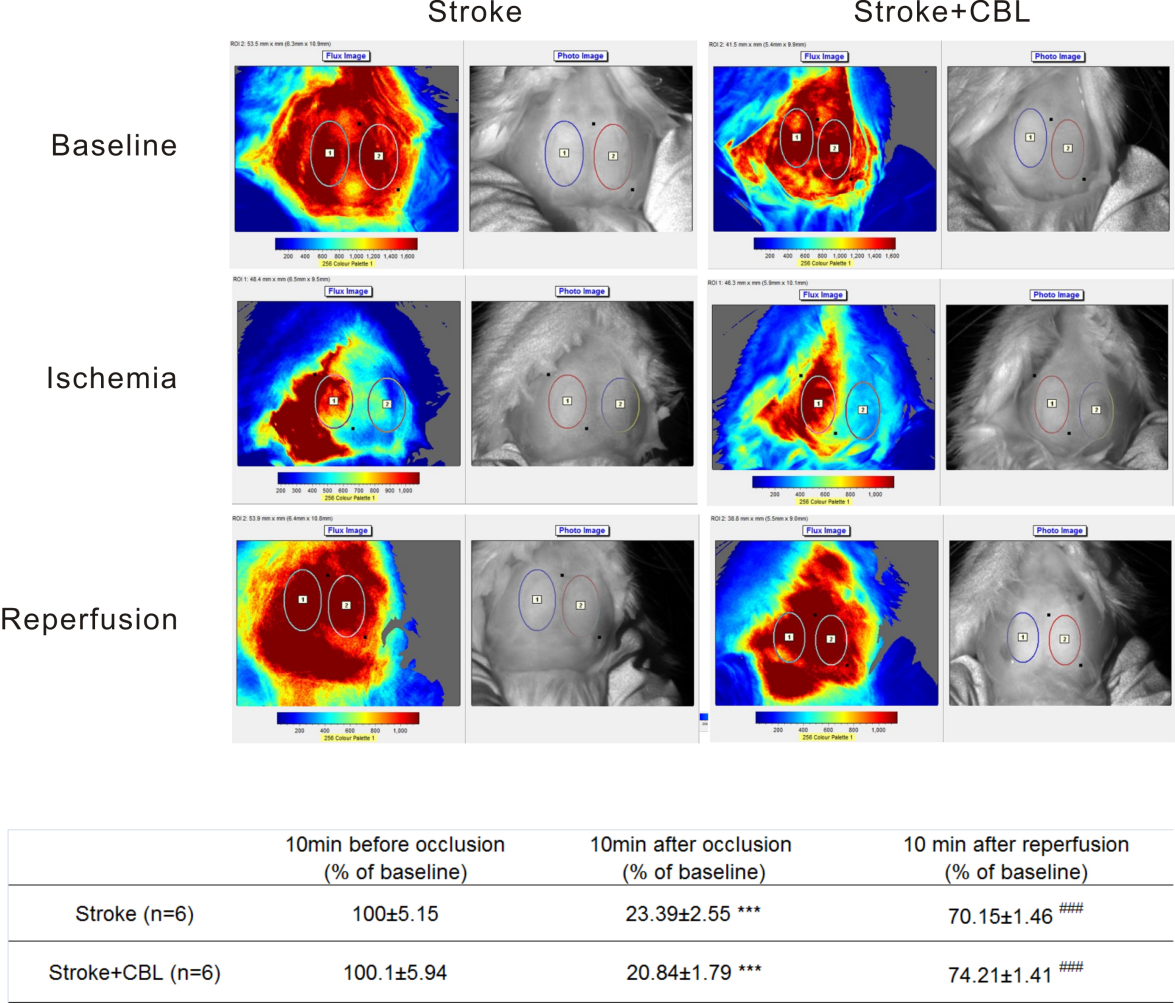


**Figure S3. Full gel for Western blotting data.
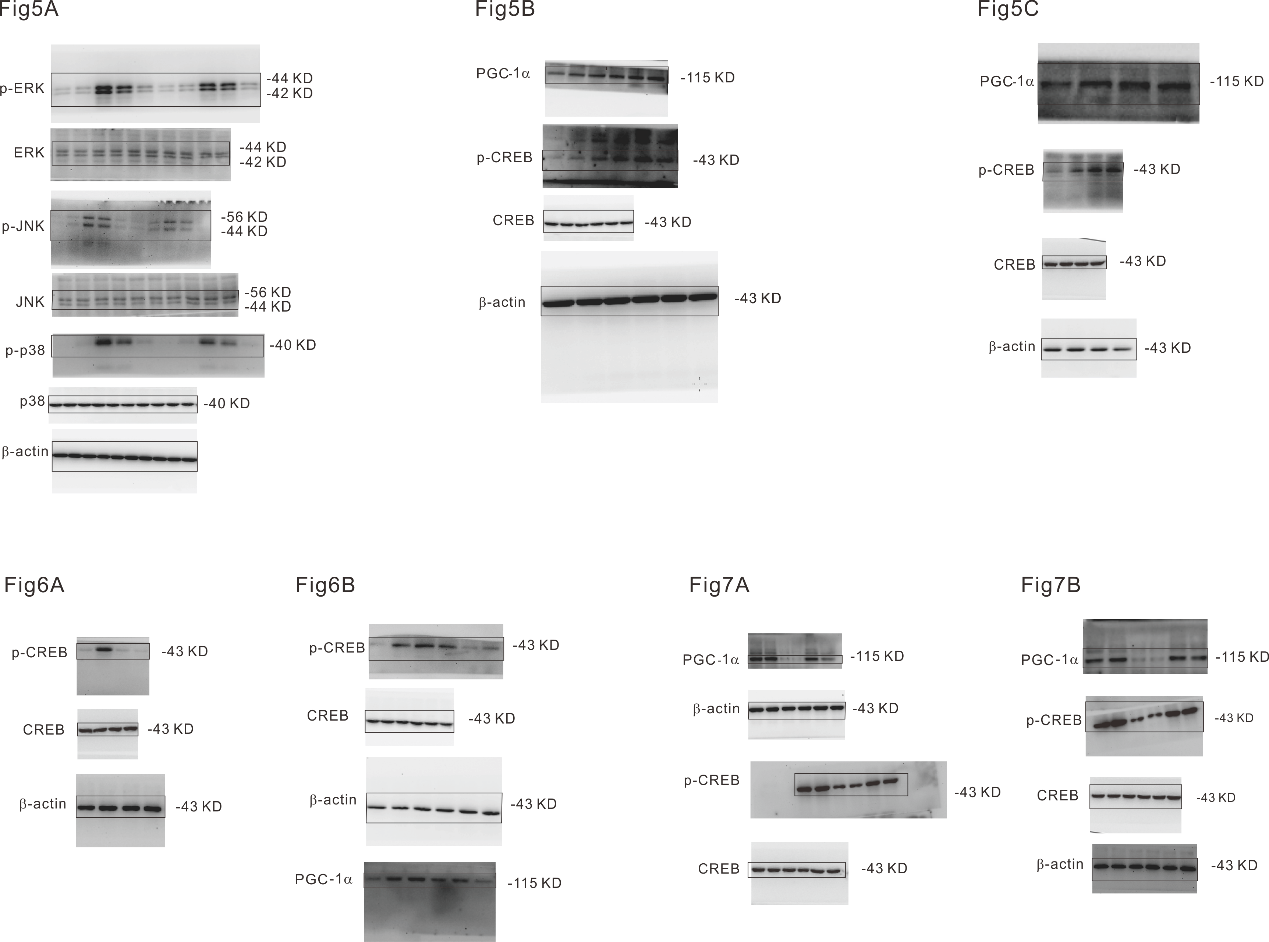
**

**Table S1. Detailed statistical analysis**

**Figure 1E TNF-α mRNA**

| One-way analysis of variance |  |
| --- | --- |
| P value | < 0.0001 |
| P value summary | *** |
| Are means signif. different? (P < 0.05) | Yes |
| Number of groups | 3 |
| F | 118 |
| R squared | 0.9633 |

| ANOVA Table | SS | df | MS |
| --- | --- | --- | --- |
| Treatment (between columns) | 252.7 | 2 | 126.3 |
| Residual (within columns) | 9.633 | 9 | 1.07 |
| Total | 262.3 | 11 |  |

| Tukey's multiple comparisons test | Mean Diff. | 95.00% CI of diff. | Significant? |
| --- | --- | --- | --- |
|  |  |  |  |
| Sham vs. Stroke | -11.17 | -13.21 to -9.128 | Yes |
| Sham vs. Stroke+CBL | -6.667 | -8.71 to -4.625 | Yes |
| Stroke vs. Stroke+CBL | 4.503 | 2.461 to 6.546 | Yes |

**Figure 1E IL-1β mRNA**

| One-way analysis of variance |  |
| --- | --- |
| P value | < 0.0001 |
| P value summary | *** |
| Are means signif. different? (P < 0.05) | Yes |
| Number of groups | 3 |
| F | 177.2 |
| R squared | 0.9752 |

| ANOVA Table | SS | df | MS |
| --- | --- | --- | --- |
| Treatment (between columns) | 252.7 | 2 | 126.3 |
| Residual (within columns) | 9.633 | 9 | 1.07 |
| Total | 262.3 | 11 |  |

| Tukey's multiple comparisons test | Mean Diff. | 95.00% CI of diff. | Significant? |
| --- | --- | --- | --- |
|  |  |  |  |
| Sham vs. Stroke | -11.17 | -13.21 to -9.128 | Yes |
| Sham vs. Stroke+CBL | -6.667 | -8.71 to -4.625 | Yes |
| Stroke vs. Stroke+CBL | 4.503 | 2.461 to 6.546 | Yes |

**Figure 1E iNOS mRNA**

| One-way analysis of variance |  |  |
| --- | --- | --- |
| P value | < 0.0001 |  |
| P value summary | *** |  |
| Are means signif. different? (P < 0.05) | Yes |  |
| Number of groups | 3 |  |
| F | 67.01 |  |
| R squared | 0.9371 |  |

| ANOVA Table | SS | df | MS |
| --- | --- | --- | --- |
| Treatment (between columns) | 29.29 | 2 | 14.64 |
| Residual (within columns) | 1.967 | 9 | 0.2185 |
| Total | 31.25 | 11 |  |

| Tukey's multiple comparisons test | Mean Diff. | 95.00% CI of diff. | Significant? |
| --- | --- | --- | --- |
|  |  |  |  |
| Sham vs. Stroke | -3.818 | -4.741 to -2.895 | Yes |
| Sham vs. Stroke+CBL | -1.684 | -2.607 to -0.7614 | Yes |
| Stroke vs. Stroke+CBL | 2.134 | 1.211 to 3.056 | Yes |

**Figure 1F CD206 mRNA**

| One-way analysis of variance |  |  |
| --- | --- | --- |
| P value | < 0.0001 |  |
| P value summary | *** |  |
| Are means signif. different? (P < 0.05) | Yes |  |
| Number of groups | 3 |  |
| F | 2839 |  |
| R squared | 0.9984 |  |

| ANOVA Table | SS | df | MS |
| --- | --- | --- | --- |
| Treatment (between columns) | 9875 | 2 | 4937 |
| Residual (within columns) | 15.65 | 9 | 1.739 |
| Total | 9891 | 11 |  |

| Tukey's multiple comparisons test | Mean Diff. | 95.00% CI of diff. | Significant? |
| --- | --- | --- | --- |
|  |  |  |  |
| Sham vs. Stroke | -3.429 | -6.033 to -0.8257 | Yes |
| Sham vs. Stroke+CBL | -62.49 | -65.1 to -59.89 | Yes |
| Stroke vs. Stroke+CBL | -59.07 | -61.67 to -56.46 | Yes |

**Figure 1F YM1/2 mRNA**

| One-way analysis of variance |  |  |
| --- | --- | --- |
| P value | < 0.0001 |  |
| P value summary | *** |  |
| Are means signif. different? (P < 0.05) | Yes |  |
| Number of groups | 3 |  |
| F | 314.2 |  |
| R squared | 0.9859 |  |

| ANOVA Table | SS | df | MS |
| --- | --- | --- | --- |
| Treatment (between columns) | 82.06 | 2 | 41.03 |
| Residual (within columns) | 1.175 | 9 | 0.1306 |
| Total | 83.23 | 11 |  |

| Tukey's multiple comparisons test | Mean Diff. | 95.00% CI of diff. | Significant? |
| --- | --- | --- | --- |
|  |  |  |  |
| Sham vs. Stroke | -3.397 | -4.11 to -2.684 | Yes |
| Sham vs. Stroke+CBL | -6.401 | -7.115 to -5.688 | Yes |
| Stroke vs. Stroke+CBL | -3.004 | -3.718 to -2.291 | Yes |

**Figure 1F Arginase 1 mRNA**

| One-way analysis of variance |  |  |
| --- | --- | --- |
| P value | < 0.0001 |  |
| P value summary | *** |  |
| Are means signif. different? (P < 0.05) | Yes |  |
| Number of groups | 3 |  |
| F | 414.6 |  |
| R squared | 0.9893 |  |

| ANOVA Table | SS | df | MS |
| --- | --- | --- | --- |
| Treatment (between columns) | 16.35 | 2 | 8.177 |
| Residual (within columns) | 0.1775 | 9 | 0.01972 |
| Total | 16.53 | 11 |  |

| Tukey's multiple comparisons test | Mean Diff. | 95.00% CI of diff. | Significant? |
| --- | --- | --- | --- |
|  |  |  |  |
| Sham vs. Stroke | -1.694 | -1.972 to -1.417 | Yes |
| Sham vs. Stroke+CBL | -2.842 | -3.119 to -2.565 | Yes |
| Stroke vs. Stroke+CBL | -1.148 | -1.425 to -0.8703 | Yes |

**Figure 2B Survival proportions**

| Log-rank(Mantel-Cox) test | P value. | Significant? |
| --- | --- | --- |
| Stroke vs. Stroke+CBL | 0.0301 | Yes |
| Gehan-Breslow-Wilcoxon test | P value. | Significant? |
| Stroke vs. Stroke+CBL | 0.0466 | Yes |

**Figure 3B total distance**

| One-way analysis of variance |  |  |
| --- | --- | --- |
| P value | < 0.0001 |  |
| P value summary | *** |  |
| Are means signif. different? (P < 0.05) | Yes |  |
| Number of groups | 5 |  |
| F | 99.4 |  |
| R squared | 0.9191 |  |

| ANOVA Table | SS | df | MS |
| --- | --- | --- | --- |
| Treatment (between columns) | 2013 | 4 | 503.4 |
| Residual (within columns) | 177.2 | 35 | 5.064 |
| Total | 2191 | 39 |  |

| Dunnett's multiple comparisons test | Mean Diff. | 95.00% CI of diff. | Significant? |
| --- | --- | --- | --- |
|  |  |  |  |
| LPS vs. CN | -21.45 | -24.33 to -18.58 | Yes |
| LPS vs. 20 | -5.28 | -8.158 to -2.401 | Yes |
| CN vs. 60 | 11.46 | 8.577 to 14.33 | Yes |
| CN vs. 100 | 11.23 | 8.356 to 14.11 | Yes |

**Figure 3B central distance**

| One-way analysis of variance |  |  |
| --- | --- | --- |
| P value | < 0.0001 |  |
| P value summary | *** |  |
| Are means signif. different? (P < 0.05) | Yes |  |
| Number of groups | 5 |  |
| F | 50.88 |  |
| R squared | 0.8715 |  |

| ANOVA Table | SS | df | MS |
| --- | --- | --- | --- |
| Treatment (between columns) | 101.4 | 4 | 25.36 |
| Residual (within columns) | 14.95 | 30 | 0.4984 |
| Total | 116.4 | 34 |  |

| Dunnett's multiple comparisons test | Mean Diff. | 95.00% CI of diff. | Significant? |
| --- | --- | --- | --- |
|  |  |  |  |
| LPS vs. CN | 4.955 | 3.982 to 5.928 | Yes |
| LPS vs. 20 | 3.674 | 2.701 to 4.647 | Yes |
| CN vs. 60 | 1.998 | 1.025 to 2.971 | Yes |
| CN vs. 100 | 1.736 | 0.7626 to 2.709 | Yes |

**Figure 3B time towards central area**

| One-way analysis of variance |  |  |
| --- | --- | --- |
| P value | < 0.0001 |  |
| P value summary | *** |  |
| Are means signif. different? (P < 0.05) | Yes |  |
| Number of groups | 5 |  |
| F | 63.33 |  |
| R squared | 0.9005 |  |

| ANOVA Table | SS | df | MS |
| --- | --- | --- | --- |
| Treatment (between columns) | 30777 | 4 | 7694 |
| Residual (within columns) | 3402 | 28 | 121.5 |
| Total | 34179 | 32 |  |

| Dunnett's multiple comparisons test | Mean Diff. | 95.00% CI of diff. | Significant? |
| --- | --- | --- | --- |
|  |  |  |  |
| LPS vs. CN | -69.6 | -85.01 to -54.19 | Yes |
| LPS vs. 20 | -37.5 | -52.91 to -22.09 | Yes |
| LPS vs. 60 | -68.9 | -83.67 to -54.13 | Yes |
| LPS vs. 100 | -79.18 | -94.59 to -63.77 | Yes |

**Figure 3B line crossings**

| One-way analysis of variance |  |  |
| --- | --- | --- |
| P value | < 0.0001 |  |
| P value summary | *** |  |
| Are means signif. different? (P < 0.05) | Yes |  |
| Number of groups | 5 |  |
| F | 95.74 |  |
| R squared | 0.9141 |  |

| ANOVA Table | SS | df | MS |
| --- | --- | --- | --- |
| Treatment (between columns) | 104990 | 4 | 26247 |
| Residual (within columns) | 9869 | 36 | 274.2 |
| Total | 114859 | 40 |  |
| Dunnett's multiple comparisons test | Mean Diff. | 95.00% CI of diff. | Significant? |
|  |  |  |  |
| LPS vs. CN | -163.6 | -185.8 to -141.3 | Yes |
| LPS vs. 20 | -49.03 | -70.35 to -27.71 | Yes |
| LPS vs. 60 | -85.98 | -105 to -66.97 | Yes |
| LPS vs. 100 | -88.64 | -109.2 to -68.08 | Yes |

| \|  \|  \| \| --- \| --- \|   **Figure 3B time in the central area**   \| One-way analysis of variance \|  \| \| --- \| --- \| \| P value \| < 0.0001 \| \| P value summary \| *** \| \| Are means signif. different? (P < 0.05) \| Yes \| \| Number of groups \| 5 \| \| F \| 60.99 \|  \| \| R squared \| 0.8905 \|  \| ANOVA Table \| SS \| df \| MS \| \| --- \| --- \| --- \| --- \| \| Treatment (between columns) \| 6883 \| 4 \| 1721 \| \| Residual (within columns) \| 846.4 \| 30 \| 28.21 \| \| Total \| 7730 \| 34 \|  \|  \| Dunnett's multiple comparisons test \| Mean Diff. \| 95.00% CI of diff. \| Significant? \| \| --- \| --- \| --- \| --- \| \|  \|  \|  \|  \| \| LPS vs. CN \| -40.04 \| -47.26 to -32.82 \| Yes \| \| LPS vs. 20 \| -15.96 \| -23.17 to -8.738 \| Yes \| \| LPS vs. 60 \| -26.85 \| -33.51 to -20.2 \| Yes \| \| LPS vs. 100 \| -28.29 \| -35.51 to -21.07 \| Yes \|   **Figure 3B number exits central area**   \| One-way analysis of variance \|  \| \| --- \| --- \| \| P value \| < 0.0001 \| \| P value summary \| *** \| \| Are means signif. different? (P < 0.05) \| Yes \| \| Number of groups \| 5 \| \| F \| 60.33 \|  \| \| R squared \| 0.8797 \|  \| ANOVA Table \| SS \| df \| MS \| \| --- \| --- \| --- \| --- \| \| Treatment (between columns) \| 2161 \| 4 \| 540.3 \| \| Residual (within columns) \| 295.6 \| 33 \| 8.956 \| \| Total \| 2457 \| 37 \|  \|  \| Dunnett's multiple comparisons test \| Mean Diff. \| 95.00% CI of diff. \| Significant? \| \| --- \| --- \| --- \| --- \| \|  \|  \|  \|  \| \| LPS vs. CN \| -21.63 \| -25.5 to -17.77 \| Yes \| \| LPS vs. 20 \| -4.571 \| -8.675 to -0.4677 \| Yes \| \| LPS vs. 60 \| -12.29 \| -16.39 to -8.182 \| Yes \| \| LPS vs. 100 \| -11.36 \| -15.33 to -7.384 \| Yes \|   **Figure 3C TNF-α Protein**   \| One-way analysis of variance \|  \| \| --- \| --- \| \| P value \| < 0.0001 \| \| P value summary \| *** \| \| Are means signif. different? (P < 0.05) \| Yes \| \| Number of groups \| 5 \| \| F \| 73.43 \|  \| \| R squared \| 0.9245 \|  \| ANOVA Table \| SS \| df \| MS \| \| --- \| --- \| --- \| --- \| \| Treatment (between columns) \| 767.1 \| 4 \| 191.8 \| \| Residual (within columns) \| 62.69 \| 24 \| 2.612 \| \| Total \| 829.8 \| 28 \|  \|  \| Dunnett's multiple comparisons test \| Mean Diff. \| 95.00% CI of diff. \| Significant? \| \| --- \| --- \| --- \| --- \| \|  \|  \|  \|  \| \| LPS vs. CN \| 16.36 \| 13.63 to 19.08 \| Yes \| \| LPS vs. 20 \| 11.68 \| 9.036 to 14.33 \| Yes \| \| LPS vs. 60 \| 14.33 \| 11.61 to 17.06 \| Yes \| \| LPS vs. 100 \| 14.75 \| 12.02 to 17.48 \| Yes \|  \| \| **Figure 3C TNF-α mRNA**  One-way analysis of variance \|  \| \| --- \| --- \| \| P value \| < 0.0001 \| \| P value summary \| *** \| \| Are means signif. different? (P < 0.05) \| Yes \| \| Number of groups \| 5 \| \| F \| 136 \|  \| \| R squared \| 0.9527 \|  \| ANOVA Table \| SS \| df \| MS \| \| --- \| --- \| --- \| --- \| \| Treatment (between columns) \| 186.8 \| 4 \| 46.69 \| \| Residual (within columns) \| 9.269 \| 27 \| 0.3433 \| \| Total \| 196 \| 31 \|  \|  \| Dunnett's multiple comparisons test \| Mean Diff. \| 95.00% CI of diff. \| Significant? \| \| --- \| --- \| --- \| --- \| \|  \|  \|  \|  \| \| LPS vs. CN \| 6.605 \| 5.727 to 7.482 \| Yes \| \| LPS vs. 20 \| 4.915 \| 4.038 to 5.793 \| Yes \| \| LPS vs. 60 \| 6.204 \| 5.327 to 7.082 \| Yes \| \| LPS vs. 100 \| 6.273 \| 5.452 to 7.093 \| Yes \| \|  \| \| --- \| --- \| --- \| --- \| --- \| --- \| --- \| --- \| --- \| --- \| --- \| --- \| --- \| --- \| --- \| --- \| --- \| --- \| --- \| --- \| --- \| --- \| --- \| --- \| --- \| --- \| --- \| --- \| --- \| --- \| --- \| --- \| --- \| --- \| --- \| --- \| --- \| --- \| --- \| --- \| --- \| --- \| --- \| --- \| --- \| --- \| --- \| --- \| --- \| --- \| --- \| --- \| --- \| --- \| --- \| --- \| --- \| |  |
| --- | --- | --- | --- | --- | --- | --- | --- | --- | --- | --- | --- | --- | --- | --- | --- | --- | --- | --- | --- | --- | --- | --- | --- | --- | --- | --- | --- | --- | --- | --- | --- | --- | --- | --- | --- | --- | --- | --- | --- | --- | --- | --- | --- | --- | --- | --- | --- | --- | --- | --- | --- | --- | --- | --- | --- | --- | --- | --- | --- | --- | --- | --- | --- | --- | --- | --- | --- | --- | --- | --- | --- | --- | --- | --- | --- | --- | --- | --- | --- | --- | --- | --- | --- | --- | --- | --- | --- | --- | --- | --- | --- | --- | --- | --- | --- | --- | --- | --- | --- | --- | --- | --- | --- | --- | --- | --- | --- | --- | --- | --- | --- | --- | --- | --- | --- | --- | --- | --- | --- | --- | --- | --- | --- | --- | --- | --- | --- | --- | --- | --- | --- | --- | --- | --- | --- | --- | --- | --- | --- | --- | --- | --- | --- | --- | --- | --- | --- | --- | --- | --- | --- | --- | --- | --- | --- | --- | --- | --- | --- | --- | --- | --- | --- | --- | --- | --- | --- | --- | --- | --- | --- | --- | --- | --- | --- | --- | --- | --- | --- | --- | --- | --- | --- | --- | --- | --- | --- | --- | --- | --- | --- | --- | --- | --- | --- | --- | --- | --- | --- | --- | --- | --- | --- | --- | --- | --- | --- | --- | --- | --- | --- | --- | --- | --- | --- | --- | --- | --- | --- | --- | --- | --- | --- | --- | --- |

| **Figure 3C iNOS mRNA**  One-way analysis of variance |  |  |
| --- | --- | --- |
| P value | < 0.0001 |  |
| P value summary | *** |  |
| Are means signif. different? (P < 0.05) | Yes |  |
| Number of groups | 5 |  |
| F | 98.79 |  |
| R squared | 0.9518 |  |

| ANOVA Table | SS | df | MS |
| --- | --- | --- | --- |
| Treatment (between columns) | 3451 | 4 | 862.9 |
| Residual (within columns) | 174.7 | 20 | 8.735 |
| Total | 3626 | 24 |  |

| Dunnett's multiple comparisons test | Mean Diff. | 95.00% CI of diff. | Significant? |
| --- | --- | --- | --- |
|  |  |  |  |
| LPS vs. CN | 33.88 | 28.92 to 38.83 | Yes |
| LPS vs. 20 | 20.55 | 15.59 to 25.51 | Yes |
| LPS vs. 60 | 27.98 | 23.02 to 32.93 | Yes |
| LPS vs. 100 | 27.24 | 22.28 to 32.19 | Yes |

| **Figure 3C COX2 mRNA**  One-way analysis of variance |  |  |
| --- | --- | --- |
| P value | < 0.0001 |  |
| P value summary | *** |  |
| Are means signif. different? (P < 0.05) | Yes |  |
| Number of groups | 5 |  |
| F | 181.8 |  |
| R squared | 0.9732 |  |

| ANOVA Table | SS | df | MS |
| --- | --- | --- | --- |
| Treatment (between columns) | 1383 | 4 | 345.7 |
| Residual (within columns) | 38.03 | 20 | 1.901 |
| Total | 1421 | 24 |  |

| Dunnett's multiple comparisons test | Mean Diff. | 95.00% CI of diff. | Significant? |
| --- | --- | --- | --- |
|  |  |  |  |
| LPS vs. CN | 22.42 | 20.11 to 24.73 | Yes |
| LPS vs. 20 | 5.105 | 2.793 to 7.417 | Yes |
| LPS vs. 60 | 9.888 | 7.576 to 12.2 | Yes |
| LPS vs. 100 | 9.519 | 7.207 to 11.83 | Yes |

| **Figure 3C CD206 mRNA**  One-way analysis of variance |  |  |
| --- | --- | --- |
| P value | < 0.0001 |  |
| P value summary | *** |  |
| Are means signif. different? (P < 0.05) | Yes |  |
| Number of groups | 5 |  |
| F | 31.19 |  |
| R squared | 0.8863 |  |

| ANOVA Table | SS | df | MS |
| --- | --- | --- | --- |
| Treatment (between columns) | 44.19 | 4 | 11.05 |
| Residual (within columns) | 5.668 | 16 | 0.3543 |
| Total | 49.86 | 20 |  |

| Dunnett's multiple comparisons test | Mean Diff. | 95.00% CI of diff. | Significant? |
| --- | --- | --- | --- |
|  |  |  |  |
| LPS vs. CN | 1.175 | 0.03554 to 2.315 | Yes |
| LPS vs. 20 | -0.3038 | -1.444 to 0.836 | No |
| LPS vs. 60 | -2.42 | -3.501 to -1.339 | Yes |
| LPS vs. 100 | -2.539 | -3.679 to -1.399 | Yes |

| **Figure 3C IL-10 mRNA**  One-way analysis of variance |  |  |
| --- | --- | --- |
| P value | < 0.0001 |  |
| P value summary | *** |  |
| Are means signif. different? (P < 0.05) | Yes |  |
| Number of groups | 5 |  |
| F | 54.36 |  |
| R squared | 0.9006 |  |

| ANOVA Table | SS | df | MS |
| --- | --- | --- | --- |
| Treatment (between columns) | 20.28 | 4 | 5.071 |
| Residual (within columns) | 2.239 | 24 | 0.09329 |
| Total | 22.52 | 28 |  |

| Dunnett's multiple comparisons test | Mean Diff. | 95.00% CI of diff. | Significant? |
| --- | --- | --- | --- |
|  |  |  |  |
| LPS vs. CN | 0.0061 | -0.4774 to 0.4896 | No |
| LPS vs. 20 | -0.5932 | -1.054 to -0.1321 | Yes |
| LPS vs. 60 | -1.927 | -2.388 to -1.466 | Yes |
| LPS vs. 100 | -1.76 | -2.221 to -1.299 | Yes |

| **Figure 3C Arginase 1 mRNA**  One-way analysis of variance |  |  |
| --- | --- | --- |
| P value | < 0.0001 |  |
| P value summary | *** |  |
| Are means signif. different? (P < 0.05) | Yes |  |
| Number of groups | 5 |  |
| F | 233.4 |  |
| R squared | 0.9831 |  |

| ANOVA Table | SS | df | MS |
| --- | --- | --- | --- |
| Treatment (between columns) | 44.63 | 4 | 11.16 |
| Residual (within columns) | 0.765 | 16 | 0.04781 |
| Total | 45.4 | 20 |  |

| Dunnett's multiple comparisons test | Mean Diff. | 95.00% CI of diff. | Significant? |
| --- | --- | --- | --- |
|  |  |  |  |
| LPS vs. CN | 1.53 | 1.133 to 1.927 | Yes |
| LPS vs. 20 | -1.336 | -1.755 to -0.9175 | Yes |
| LPS vs. 60 | -2.137 | -2.556 to -1.718 | Yes |
| LPS vs. 100 | -2.108 | -2.527 to -1.69 | Yes |

| **Figure 4A LDH**  One-way analysis of variance |  |  |
| --- | --- | --- |
| P value | < 0.0001 |  |
| P value summary | *** |  |
| Are means signif. different? (P < 0.05) | Yes |  |
| Number of groups | 5 |  |
| F | 0.6285 |  |
| R squared | 0.2009 |  |

| ANOVA Table | SS | df | MS |
| --- | --- | --- | --- |
| Treatment (between columns) | 0.0006184 | 4 | 0.0001546 |
| Residual (within columns) | 0.00246 | 10 | 0.000246 |
| Total | 0.003078 | 14 |  |

| Dunnett's multiple comparisons test | Mean Diff. | 95.00% CI of diff. | Significant? |
| --- | --- | --- | --- |
|  |  |  |  |
| CN vs. 0.5 | -0.004864 | -0.04188 to 0.03215 | No |
| CN vs. 1 | -0.01192 | -0.04894 to 0.02509 | No |
| CN vs. 2 | 0.003485 | -0.03353 to 0.0405 | No |
| CN vs. 5 | 0.006186 | -0.03083 to 0.0432 | No |

| **Figure 4B TNF-α protein**  One-way analysis of variance |  |  |
| --- | --- | --- |
| P value | < 0.0001 |  |
| P value summary | *** |  |
| Are means signif. different? (P < 0.05) | Yes |  |
| Number of groups | 6 |  |
| F | 218.1 |  |
| R squared | 0.9891 |  |

| ANOVA Table | SS | df | MS |
| --- | --- | --- | --- |
| Treatment (between columns) | 111.4 | 5 | 22.28 |
| Residual (within columns) | 1.226 | 12 | 0.1022 |
| Total | 112.6 | 17 |  |

| Dunnett's multiple comparisons test | Mean Diff. | 95.00% CI of diff. | Significant? |
| --- | --- | --- | --- |
|  |  |  |  |
| LPS vs. CN | 7.464 | 6.707 to 8.221 | Yes |
| LPS vs. 0.5 | 0.2673 | -0.49 to 1.025 | No |
| LPS vs. 1 | 1.558 | 0.8004 to 2.315 | Yes |
| LPS vs. 2 | 2.231 | 1.474 to 2.988 | Yes |
| LPS vs. 5 | 3.074 | 2.316 to 3.831 | Yes |

| **Figure 4C TNF-α mRNA**  One-way analysis of variance |  |  |
| --- | --- | --- |
| P value | < 0.0001 |  |
| P value summary | *** |  |
| Are means signif. different? (P < 0.05) | Yes |  |
| Number of groups | 6 |  |
| F | 43.43 |  |
| R squared | 0.9476 |  |

| ANOVA Table | SS | df | MS |
| --- | --- | --- | --- |
| Treatment (between columns) | 1.907 | 5 | 0.3815 |
| Residual (within columns) | 0.1054 | 12 | 0.008783 |
| Total | 2.013 | 17 |  |

| Dunnett's multiple comparisons test | Mean Diff. | 95.00% CI of diff. | Significant? |
| --- | --- | --- | --- |
|  |  |  |  |
| LPS vs. CN | 0.9995 | 0.7775 to 1.221 | Yes |
| LPS vs. 0.5 | 0.3358 | 0.1138 to 0.5578 | Yes |
| LPS vs. 1 | 0.7021 | 0.4801 to 0.9241 | Yes |
| LPS vs. 2 | 0.7151 | 0.4931 to 0.9371 | Yes |
| LPS vs. 5 | 0.7553 | 0.5333 to 0.9773 | Yes |

| **Figure 4C IL-1β mRNA**  One-way analysis of variance |  |  |
| --- | --- | --- |
| P value | < 0.0001 |  |
| P value summary | *** |  |
| Are means signif. different? (P < 0.05) | Yes |  |
| Number of groups | 6 |  |
| F | 69.67 |  |
| R squared | 0.9667 |  |

| ANOVA Table | SS | df | MS |
| --- | --- | --- | --- |
| Treatment (between columns) | 1.764 | 5 | 0.3528 |
| Residual (within columns) | 0.06077 | 12 | 0.005064 |
| Total | 1.825 | 17 |  |

| Dunnett's multiple comparisons test | Mean Diff. | 95.00% CI of diff. | Significant? |
| --- | --- | --- | --- |
|  |  |  |  |
| LPS vs. CN | 0.9961 | 0.8276 to 1.165 | Yes |
| LPS vs. 0.5 | 0.3019 | 0.1333 to 0.4705 | Yes |
| LPS vs. 1 | 0.6844 | 0.5159 to 0.853 | Yes |
| LPS vs. 2 | 0.5617 | 0.3931 to 0.7302 | Yes |
| LPS vs. 5 | 0.6439 | 0.4753 to 0.8124 | Yes |

| **Figure 4C COX2 mRNA**  One-way analysis of variance |  |  |
| --- | --- | --- |
| P value | < 0.0001 |  |
| P value summary | *** |  |
| Are means signif. different? (P < 0.05) | Yes |  |
| Number of groups | 6 |  |
| F | 212.1 |  |
| R squared | 0.9881 |  |

| ANOVA Table | SS | df | MS |
| --- | --- | --- | --- |
| Treatment (between columns) | 1.978 | 5 | 0.3957 |
| Residual (within columns) | 0.02239 | 12 | 0.001866 |
| Total | 2.001 | 17 |  |

| Dunnett's multiple comparisons test | Mean Diff. | 95.00% CI of diff. | Significant? |
| --- | --- | --- | --- |
|  |  |  |  |
| LPS vs. CN | 0.9429 | 0.8406 to 1.045 | Yes |
| LPS vs. 0.5 | -0.04502 | -0.1473 to 0.0573 | No |
| LPS vs. 1 | 0.2816 | 0.1793 to 0.3839 | Yes |
| LPS vs. 2 | 0.3972 | 0.2949 to 0.4995 | Yes |
| LPS vs. 5 | 0.5002 | 0.3979 to 0.6025 | Yes |

| **Figure 4C iNOS mRNA**  One-way analysis of variance |  |  |
| --- | --- | --- |
| P value | < 0.0001 |  |
| P value summary | *** |  |
| Are means signif. different? (P < 0.05) | Yes |  |
| Number of groups | 6 |  |
| F | 62.04 |  |
| R squared | 0.9628 |  |

| ANOVA Table | SS | df | MS |
| --- | --- | --- | --- |
| Treatment (between columns) | 1.907 | 5 | 0.3813 |
| Residual (within columns) | 0.07376 | 12 | 0.006146 |
| Total | 1.98 | 17 |  |

| Dunnett's multiple comparisons test | Mean Diff. | 95.00% CI of diff. | Significant? |
| --- | --- | --- | --- |
|  |  |  |  |
| LPS vs. CN | 0.9379 | 0.7522 to 1.124 | Yes |
| LPS vs. 0.5 | 0.0547 | -0.131 to 0.2404 | No |
| LPS vs. 1 | 0.2979 | 0.1122 to 0.4836 | Yes |
| LPS vs. 2 | 0.5846 | 0.3989 to 0.7703 | Yes |
| LPS vs. 5 | 0.5503 | 0.3646 to 0.7361 | Yes |

| **Figure 4C CD206 mRNA**  One-way analysis of variance |  |  |
| --- | --- | --- |
| P value | < 0.0001 |  |
| P value summary | *** |  |
| Are means signif. different? (P < 0.05) | Yes |  |
| Number of groups | 6 |  |
| F | 47.51 |  |
| R squared | 0.9519 |  |

| ANOVA Table | SS | df | MS |
| --- | --- | --- | --- |
| Treatment (between columns) | 34.96 | 5 | 6.993 |
| Residual (within columns) | 1.766 | 12 | 0.1472 |
| Total | 36.73 | 17 |  |

| Dunnett's multiple comparisons test | Mean Diff. | 95.00% CI of diff. | Significant? |
| --- | --- | --- | --- |
|  |  |  |  |
| LPS vs. CN | -3.869 | -4.777 to -2.96 | Yes |
| LPS vs. 0.5 | 0.01121 | -0.8977 to 0.9201 | No |
| LPS vs. 1 | -0.06173 | -0.9706 to 0.8471 | No |
| LPS vs. 2 | -0.362 | -1.271 to 0.5469 | No |
| LPS vs. 5 | -1.37 | -2.278 to -0.4607 | Yes |

| **Figure 4C Arginase 1 mRNA**  One-way analysis of variance |  |  |
| --- | --- | --- |
| P value | < 0.0001 |  |
| P value summary | *** |  |
| Are means signif. different? (P < 0.05) | Yes |  |
| Number of groups | 6 |  |
| F | 8.602 |  |
| R squared | 0.9519 |  |

| ANOVA Table | SS | df | MS |
| --- | --- | --- | --- |
| Treatment (between columns) | 5.477 | 5 | 1.095 |
| Residual (within columns) | 1.528 | 12 | 0.1273 |
| Total | 7.005 | 17 |  |

| Dunnett's multiple comparisons test | Mean Diff. | 95.00% CI of diff. | Significant? |
| --- | --- | --- | --- |
|  |  |  |  |
| LPS vs. CN | -1.151 | -1.996 to -0.3058 | Yes |
| LPS vs. 0.5 | -0.4576 | -1.303 to 0.3877 | No |
| LPS vs. 1 | -0.5333 | -1.379 to 0.3121 | No |
| LPS vs. 2 | -0.974 | -1.819 to -0.1287 | Yes |
| LPS vs. 5 | -1.719 | -2.564 to -0.8733 | Yes |

| **Figure 4C IL-10 mRNA**  One-way analysis of variance |  |  |
| --- | --- | --- |
| P value | < 0.0001 |  |
| P value summary | *** |  |
| Are means signif. different? (P < 0.05) | Yes |  |
| Number of groups | 6 |  |
| F | 18.79 |  |
| R squared | 0.8868 |  |

| ANOVA Table | SS | df | MS |
| --- | --- | --- | --- |
| Treatment (between columns) | 3.695 | 5 | 0.7391 |
| Residual (within columns) | 0.4719 | 12 | 0.03933 |
| Total | 4.167 | 17 |  |

| Dunnett's multiple comparisons test | Mean Diff. | 95.00% CI of diff. | Significant? |
| --- | --- | --- | --- |
|  |  |  |  |
| LPS vs. CN | 0.7009 | 0.2311 to 1.171 | Yes |
| LPS vs. 0.5 | 0.1923 | -0.2775 to 0.6621 | No |
| LPS vs. 1 | 0.1765 | -0.2933 to 0.6463 | No |
| LPS vs. 2 | 0.002107 | -0.4677 to 0.4719 | No |
| LPS vs. 5 | -0.8261 | -1.296 to -0.3563 | Yes |

| **Figure 5A p-ERK Protein**   \| Unpaired t test \| P value. \| Significant? \| \| --- \| --- \| --- \| \|  \|  \|  \| \| 0h LPS(100 ng/mL) vs CBL(5μg/mL)+LPS \| 0.0523 \| No \| \| 0.5 h LPS(100 ng/mL) vs CBL(5μg/mL)+LPS \| 0.1227 \| No \| \| 1h LPS(100 ng/mL) vs CBL(5μg/mL)+LPS \| 0.0549 \| No \| \| 2h LPS(100 ng/mL) vs CBL(5μg/mL)+LPS \| 0.5392 \| No \| \| 3h LPS(100 ng/mL) vs CBL(5μg/mL)+LPS \| -0.7814 \| No \|   **Figure 5A p-JNK Protein**   \| Unpaired t test \| P value. \| Significant? \| \| --- \| --- \| --- \| \|  \|  \|  \| \| 0h LPS(100 ng/mL) vs CBL(5μg/mL)+LPS \| 0.6279 \| No \| \| 0.5 h LPS(100 ng/mL) vs CBL(5μg/mL)+LPS \| 0.3660 \| No \| \| 1h LPS(100 ng/mL) vs CBL(5μg/mL)+LPS \| 0.0109 \| Yes \| \| 2h LPS(100 ng/mL) vs CBL(5μg/mL)+LPS \| 0.0104 \| Yes \| \| 3h LPS(100 ng/mL) vs CBL(5μg/mL)+LPS \| 0.9364 \| No \|     **Figure 5A p-p38 Protein**   \| Unpaired t test \| P value. \| Significant? \| \| --- \| --- \| --- \| \|  \|  \|  \| \| 0h LPS(100 ng/mL) vs CBL(5μg/mL)+LPS \| 0.1289 \| No \| \| 0.5 h LPS(100 ng/mL) vs CBL(5μg/mL)+LPS \| 0.5226 \| No \| \| 1h LPS(100 ng/mL) vs CBL(5μg/mL)+LPS \| 0.003 \| Yes \| \| 2h LPS(100 ng/mL) vs CBL(5μg/mL)+LPS \| 0.004 \| Yes \| \| 3h LPS(100 ng/mL) vs CBL(5μg/mL)+LPS \| 0.1314 \| No \|   **Figure 5B PGC-1α Protein** | |  | | |
| --- | --- | --- | --- | --- | --- | --- | --- | --- | --- | --- | --- | --- | --- | --- | --- | --- | --- | --- | --- | --- | --- | --- | --- | --- | --- | --- | --- | --- | --- | --- | --- | --- | --- | --- | --- | --- | --- | --- | --- | --- | --- | --- | --- | --- | --- | --- | --- | --- | --- | --- | --- | --- | --- | --- | --- | --- | --- | --- | --- | --- | --- | --- | --- | --- | --- | --- | --- |
| One-way analysis of variance |  | |  |  |
| P value | < 0.0001 | |  |  |
| P value summary | *** | |  |  |
| Are means signif. different? (P < 0.05) | Yes | |  |  |
| Number of groups | 6 | |  |  |
| F | 61.93 | |  |  |
| R squared | 0.9627 | |  |  |

| ANOVA Table | SS | df | MS |
| --- | --- | --- | --- |
| Treatment (between columns) | 3.831 | 5 | 0.7661 |
| Residual (within columns) | 0.1485 | 12 | 0.01237 |
| Total | 3.979 | 17 |  |

| Dunnett's multiple comparisons test | Mean Diff. | 95.00% CI of diff. | Significant? |
| --- | --- | --- | --- |
|  |  |  |  |
| 0 vs. 1 | -0.2227 | -0.4862 to 0.04076 | No |
| 0 vs. 3 | -0.5429 | -0.8064 to -0.2794 | Yes |
| 0 vs. 6 | -0.8307 | -1.094 to -0.5672 | Yes |
| 0 vs. 9 | -1.228 | -1.492 to -0.9649 | Yes |
| 0 vs. 12 | -1.194 | -1.457 to -0.9301 | Yes |

| **Figure 5B p-CREB Protein**  One-way analysis of variance |  |  |
| --- | --- | --- |
| P value | < 0.0001 |  |
| P value summary | *** |  |
| Are means signif. different? (P < 0.05) | Yes |  |
| Number of groups | 6 |  |
| F | 94.48 |  |
| R squared | 0.9752 |  |

| ANOVA Table | SS | df | MS |
| --- | --- | --- | --- |
| Treatment (between columns) | 4.43 | 5 | 0.8861 |
| Residual (within columns) | 0.1125 | 12 | 0.009378 |
| Total | 4.543 | 17 |  |

| Dunnett's multiple comparisons test | Mean Diff. | 95.00% CI of diff. | Significant? |
| --- | --- | --- | --- |
|  |  |  |  |
| 0 vs. 1 | -0.1728 | -0.4022 to 0.05665 | No |
| 0 vs. 3 | -0.5457 | -0.7751 to -0.3163 | Yes |
| 0 vs. 6 | -0.9627 | -1.192 to -0.7333 | Yes |
| 0 vs. 9 | -1.208 | -1.437 to -0.9783 | Yes |
| 0 vs. 12 | -1.298 | -1.528 to -1.069 | Yes |

| **Figure 5C PGC-1α Protein**  One-way analysis of variance |  |  |
| --- | --- | --- |
| P value | < 0.0001 |  |
| P value summary | *** |  |
| Are means signif. different? (P < 0.05) | Yes |  |
| Number of groups | 4 |  |
| F | 19.02 |  |
| R squared | 0.877 |  |

| ANOVA Table | SS | df | MS |
| --- | --- | --- | --- |
| Treatment (between columns) | 2.01 | 3 | 0.6699 |
| Residual (within columns) | 0.2818 | 8 | 0.03523 |
| Total | 2.291 | 11 |  |

| Dunnett's multiple comparisons test | Mean Diff. | 95.00% CI of diff. | Significant? |
| --- | --- | --- | --- |
|  |  |  |  |
| 0 vs. 1 | -0.4607 | -0.902 to -0.01941 | Yes |
| 0 vs. 2 | -0.9175 | -1.359 to -0.4762 | Yes |
| 0 vs. 5 | -1.036 | -1.477 to -0.5943 | Yes |

| **Figure 5C p-CREB Protein**  One-way analysis of variance |  |  |
| --- | --- | --- |
| P value | < 0.0001 |  |
| P value summary | *** |  |
| Are means signif. different? (P < 0.05) | Yes |  |
| Number of groups | 4 |  |
| F | 124.4 |  |
| R squared | 0.979 |  |

| ANOVA Table | SS | df | MS |
| --- | --- | --- | --- |
| Treatment (between columns) | 1.911 | 3 | 0.6371 |
| Residual (within columns) | 0.04096 | 8 | 0.00512 |
| Total | 1.952 | 11 |  |

| Dunnett's multiple comparisons test | Mean Diff. | 95.00% CI of diff. | Significant? |
| --- | --- | --- | --- |
|  |  |  |  |
| 0 vs. 1 | -0.4144 | -0.5827 to -0.2462 | Yes |
| 0 vs. 2 | -0.909 | -1.077 to -0.7407 | Yes |
| 0 vs. 5 | -0.9862 | -1.154 to -0.818 | Yes |

| **Figure 6A p-CREB Protein**  One-way analysis of variance |  |  |
| --- | --- | --- |
| P value | < 0.0001 |  |
| P value summary | *** |  |
| Are means signif. different? (P < 0.05) | Yes |  |
| Number of groups | 4 |  |
| F | 36.57 |  |
| R squared | 0.9242 |  |

| ANOVA Table | SS | df | MS |
| --- | --- | --- | --- |
| Treatment (between columns) | 4.842 | 3 | 1.614 |
| Residual (within columns) | 0.3972 | 9 | 0.04413 |
| Total | 5.239 | 12 |  |

| Tukey's multiple comparisons test | Mean Diff. | 95.00% CI of diff. | Significant? |
| --- | --- | --- | --- |
| CN vs. CBL | -1.523 | -2.024 to -1.022 | Yes |
| CN vs. CBL+666-15 | -0.05074 | -0.5516 to 0.4502 | No |
| CN vs. 666-15 | -0.258 | -0.7589 to 0.2429 | No |
| CBL vs. CBL+666-15 | 1.472 | 0.9366 to 2.008 | Yes |
| CBL vs. 666-15 | 1.265 | 0.7294 to 1.8 | Yes |
| CBL+666-15 vs. 666-15 | -0.2073 | -0.7428 to 0.3282 | No |

| **Figure 6B PGC-1α Protein**  One-way analysis of variance |  |  |
| --- | --- | --- |
| P value | < 0.0001 |  |
| P value summary | *** |  |
| Are means signif. different? (P < 0.05) | Yes |  |
| Number of groups | 6 |  |
| F | 26．53 |  |
| R squared | 0.9171 |  |

| ANOVA Table | SS | df | MS |
| --- | --- | --- | --- |
| Treatment (between columns) | 2.783 | 5 | 0.5567 |
| Residual (within columns) | 0.2518 | 12 | 0.02098 |
| Total | 3.035 | 17 |  |

| Tukey's multiple comparisons test | Mean Diff. | 95.00% CI of diff. | Significant? |
| --- | --- | --- | --- |
| Column A vs. Column B | -0.4545 | -0.8517 to -0.05727 | Yes |
| Column A vs. Column C | -1.291 | -1.688 to -0.8935 | Yes |
| Column A vs. Column D | -0.3341 | -0.7313 to 0.06317 | No |
| Column A vs. Column E | -0.5903 | -0.9876 to -0.1931 | Yes |
| Column A vs. Column F | -0.6908 | -1.088 to -0.2936 | Yes |
| Column B vs. Column C | -0.8362 | -1.233 to -0.439 | Yes |
| Column B vs. Column D | 0.1204 | -0.2768 to 0.5177 | No |
| Column B vs. Column E | -0.1358 | -0.5331 to 0.2614 | No |
| Column B vs. Column F | -0.2363 | -0.6336 to 0.1609 | No |
| Column C vs. Column D | 0.9567 | 0.5594 to 1.354 | Yes |
| Column C vs. Column E | 0.7004 | 0.3032 to 1.098 | Yes |
| Column C vs. Column F | 0.5999 | 0.2027 to 0.9971 | Yes |
| Column D vs. Column E | -0.2563 | -0.6535 to 0.141 | No |
| Column D vs. Column F | -0.3567 | -0.754 to 0.04049 | No |
| Column E vs. Column F | -0.1005 | -0.4977 to 0.2967 | No |

| **Figure 6B p-CREB Protein**  One-way analysis of variance |  |  |
| --- | --- | --- |
| P value | < 0.0001 |  |
| P value summary | *** |  |
| Are means signif. different? (P < 0.05) | Yes |  |
| Number of groups | 6 |  |
| F | 77.89 |  |
| R squared | 0.9701 |  |

| ANOVA Table | SS | df | MS |
| --- | --- | --- | --- |
| Treatment (between columns) | 5.68 | 5 | 1.136 |
| Residual (within columns) | 0.175 | 12 | 0.01459 |
| Total | 5.855 | 17 |  |

| Tukey's multiple comparisons test | Mean Diff. | 95.00% CI of diff. | Significant? |
| --- | --- | --- | --- |
| Column A vs. Column B | -0.7175 | -1.049 to -0.3862 | Yes |
| Column A vs. Column C | -1.659 | -1.99 to -1.327 | Yes |
| Column A vs. Column D | -0.3172 | -0.6484 to 0.01401 | No |
| Column A vs. Column E | -0.03568 | -0.3669 to 0.2955 | No |
| Column A vs. Column F | -0.6817 | -1.013 to -0.3505 | Yes |
| Column B vs. Column C | -0.9411 | -1.272 to -0.6099 | Yes |
| Column B vs. Column D | 0.4002 | 0.06901 to 0.7315 | Yes |
| Column B vs. Column E | 0.6818 | 0.3506 to 1.013 | Yes |
| Column B vs. Column F | 0.03575 | -0.2955 to 0.367 | No |
| Column C vs. Column D | 1.341 | 1.01 to 1.673 | Yes |
| Column C vs. Column E | 1.623 | 1.292 to 1.954 | Yes |
| Column C vs. Column F | 0.9769 | 0.6456 to 1.308 | Yes |
| Column D vs. Column E | 0.2815 | -0.04969 to 0.6128 | No |
| Column D vs. Column F | -0.3645 | -0.6957 to -0.03326 | Yes |
| Column E vs. Column F | -0.646 | -0.9773 to -0.3148 | Yes |

| **Figure 6C TNF-α mRNA**  One-way analysis of variance |  |  |
| --- | --- | --- |
| P value | < 0.0001 |  |
| P value summary | *** |  |
| Are means signif. different? (P < 0.05) | Yes |  |
| Number of groups | 5 |  |
| F | 131.3 |  |
| R squared | 0.9813 |  |

| ANOVA Table | SS | df | MS |
| --- | --- | --- | --- |
| Treatment (between columns) | 2.314 | 4 | 0.5785 |
| Residual (within columns) | 0.04406 | 10 | 0.004406 |
| Total | 2.358 | 14 |  |

| Tukey's multiple comparisons test | Mean Diff. | 95.00% CI of diff. | Significant? |
| --- | --- | --- | --- |
| CN vs. LPS | -0.9963 | -1.175 to -0.8179 | Yes |
| CN vs. LPS+CBL | -0.5473 | -0.7257 to -0.369 | Yes |
| CN vs. LPS+CBL+666-15 | -1.039 | -1.218 to -0.8611 | Yes |
| CN vs. LPS+666-15 | -0.9345 | -1.113 to -0.7561 | Yes |
| LPS vs. LPS+CBL | 0.449 | 0.2706 to 0.6273 | Yes |
| LPS vs. LPS+CBL+666-15 | -0.0432 | -0.2216 to 0.1352 | No |
| LPS vs. LPS+666-15 | 0.06181 | -0.1165 to 0.2402 | No |
| LPS+CBL vs. LPS+CBL+666-15 | -0.4922 | -0.6705 to -0.3138 | Yes |
| LPS+CBL vs. LPS+666-15 | -0.3871 | -0.5655 to -0.2088 | Yes |
| LPS+CBL+666-15 vs. LPS+666-15 | 0.105 | -0.07335 to 0.2834 | No |

| **Figure 6C COX2 mRNA**  One-way analysis of variance |  |  |
| --- | --- | --- |
| P value | < 0.0001 |  |
| P value summary | *** |  |
| Are means signif. different? (P < 0.05) | Yes |  |
| Number of groups | 5 |  |
| F | 69.05 |  |
| R squared | 0.9653 |  |

| ANOVA Table | SS | df | MS |
| --- | --- | --- | --- |
| Treatment (between columns) | 1.482 | 4 | 0.3706 |
| Residual (within columns) | 0.05321 | 10 | 0.005321 |
| Total | 1.536 | 14 |  |

| Tukey's multiple comparisons test | Mean Diff. | 95.00% CI of diff. | Significant? |
| --- | --- | --- | --- |
| CN vs. LPS | -0.8759 | -1.072 to -0.6799 | Yes |
| CN vs. LPS+CBL | -0.3961 | -0.5921 to -0.2 | Yes |
| CN vs. LPS+CBL+666-15 | -0.5735 | -0.7695 to -0.3775 | Yes |
| CN vs. LPS+666-15 | -0.8017 | -0.9977 to -0.6056 | Yes |
| LPS vs. LPS+CBL | 0.4798 | 0.2838 to 0.6758 | Yes |
| LPS vs. LPS+CBL+666-15 | 0.3024 | 0.1064 to 0.4984 | Yes |
| LPS vs. LPS+666-15 | 0.07422 | -0.1218 to 0.2702 | No |
| LPS+CBL vs. LPS+CBL+666-15 | -0.1774 | -0.3735 to 0.0186 | No |
| LPS+CBL vs. LPS+666-15 | -0.4056 | -0.6016 to -0.2096 | Yes |
| LPS+CBL+666-15 vs. LPS+666-15 | -0.2282 | -0.4242 to -0.03215 | Yes |

| **Figure 6C Arginase 1 mRNA**  One-way analysis of variance |  |  |
| --- | --- | --- |
| P value | < 0.0001 |  |
| P value summary | *** |  |
| Are means signif. different? (P < 0.05) | Yes |  |
| Number of groups | 5 |  |
| F | 16.03 |  |
| R squared | 0.8963 |  |

| ANOVA Table | SS | df | MS |
| --- | --- | --- | --- |
| Treatment (between columns) | 6.936 | 4 | 1.734 |
| Residual (within columns) | 1.043 | 10 | 0.1043 |
| Total | 7.979 | 14 |  |

| Tukey's multiple comparisons test | Mean Diff. | 95.00% CI of diff. | Significant? |
| --- | --- | --- | --- |
| CN vs. LPS | 1.467 | 0.599 to 2.334 | Yes |
| CN vs. LPS+CBL | 0.5091 | -0.3586 to 1.377 | No |
| CN vs. LPS+CBL+666-15 | 1.733 | 0.8658 to 2.601 | Yes |
| CN vs. LPS+666-15 | 1.576 | 0.7082 to 2.443 | Yes |
| LPS vs. LPS+CBL | -0.9576 | -1.825 to -0.08991 | Yes |
| LPS vs. LPS+CBL+666-15 | 0.2668 | -0.6009 to 1.134 | No |
| LPS vs. LPS+666-15 | 0.1091 | -0.7585 to 0.9768 | No |
| LPS+CBL vs. LPS+CBL+666-15 | 1.224 | 0.3567 to 2.092 | Yes |
| LPS+CBL vs. LPS+666-15 | 1.067 | 0.1991 to 1.934 | Yes |
| LPS+CBL+666-15 vs. LPS+666-15 | -0.1576 | -1.025 to 0.71 | No |

| **Figure 6C IL-10 mRNA**  One-way analysis of variance |  |  |
| --- | --- | --- |
| P value | < 0.0001 |  |
| P value summary | *** |  |
| Are means signif. different? (P < 0.05) | Yes |  |
| Number of groups | 5 |  |
| F | 9621 |  |
| R squared | 0.9747 |  |

| ANOVA Table | SS | df | MS |
| --- | --- | --- | --- |
| Treatment (between columns) | 11.78 | 4 | 2.945 |
| Residual (within columns) | 0.3061 | 10 | 0.03061 |
| Total | 12.09 | 14 |  |

| Tukey's multiple comparisons test | Mean Diff. | 95.00% CI of diff. | Significant? |
| --- | --- | --- | --- |
| CN vs. LPS | -0.985 | -1.455 to -0.5149 | Yes |
| CN vs. LPS+CBL | -2.566 | -3.036 to -2.096 | Yes |
| CN vs. LPS+CBL+666-15 | -0.6249 | -1.095 to -0.1547 | Yes |
| CN vs. LPS+666-15 | -0.3916 | -0.8618 to 0.0785 | No |
| LPS vs. LPS+CBL | -1.581 | -2.051 to -1.111 | Yes |
| LPS vs. LPS+CBL+666-15 | 0.3601 | -0.11 to 0.8303 | No |
| LPS vs. LPS+666-15 | 0.5934 | 0.1232 to 1.063 | Yes |
| LPS+CBL vs. LPS+CBL+666-15 | 1.941 | 1.471 to 2.411 | Yes |
| LPS+CBL vs. LPS+666-15 | 2.175 | 1.704 to 2.645 | Yes |
| LPS+CBL+666-15 vs. LPS+666-15 | 0.2332 | -0.2369 to 0.7034 | No |

| **Figure 7A PGC-1α Protein**  One-way analysis of variance |  |  |
| --- | --- | --- |
| P value | < 0.0001 |  |
| P value summary | *** |  |
| Are means signif. different? (P < 0.05) | Yes |  |
| Number of groups | 3 |  |
| F | 27.92 |  |
| R squared | 0.7883 |  |

| ANOVA Table | SS | df | MS |
| --- | --- | --- | --- |
| Treatment (between columns) | 1.195 | 2 | 0.5974 |
| Residual (within columns) | 0.3209 | 15 | 0.02139 |
| Total | 1.516 | 17 |  |

| Dunnett's multiple comparisons test | Mean Diff. | | 95.00% CI of diff. | Significant? | |  |
| --- | --- | --- | --- | --- | --- | --- |
|  |  | |  |  | |  |
| LPS vs. CN | -0.5322 | | -0.7382 to -0.3262 | Yes | |  |
| LPS vs. LPS+CBL | -0.5598 | | -0.7658 to -0.3538 | Yes | |  |
| **Figure 7A p-CREB Protein**  One-way analysis of variance | |  | | |  |  |
| P value | | < 0.0001 | | |  |  |
| P value summary | | *** | | |  |  |
| Are means signif. different? (P < 0.05) | | Yes | | |  |  |
| Number of groups | | 3 | | |  |  |
| F | | 22.95 | | |  | |
| R squared | | 0.7537 | | |  |  |

| ANOVA Table | SS | df | MS |
| --- | --- | --- | --- |
| Treatment (between columns) | 0.8557 | 2 | 0.4279 |
| Residual (within columns) | 0.2797 | 15 | 0.01865 |
| Total | 1.135 | 17 |  |

| Dunnett's multiple comparisons test | Mean Diff. | 95.00% CI of diff. | Significant? |
| --- | --- | --- | --- |
|  |  |  |  |
| LPS vs. CN | -0.3823 | -0.5746 to -0.19 | Yes |
| LPS vs. LPS+CBL | -0.5141 | -0.7064 to -0.3218 | Yes |

| **Figure 7B PGC-1α Protein**  One-way analysis of variance |  |  |
| --- | --- | --- |
| P value | < 0.0001 |  |
| P value summary | *** |  |
| Are means signif. different? (P < 0.05) | Yes |  |
| Number of groups | 3 |  |
| F | 56.02 |  |
| R squared | 0.8819 |  |

| ANOVA Table | SS | df | MS |
| --- | --- | --- | --- |
| Treatment (between columns) | 1.695 | 2 | 0.8474 |
| Residual (within columns) | 0.2269 | 15 | 0.01513 |
| Total | 1.922 | 17 |  |

| Dunnett's multiple comparisons test | Mean Diff. | | 95.00% CI of diff. | Significant? | |  |
| --- | --- | --- | --- | --- | --- | --- |
|  |  | |  |  | |  |
| Stroke vs. Sham | -0.4502 | | -0.6234 to -0.277 | Yes | |  |
| Stroke vs. Stroke+CBL | -0.7463 | | -0.9195 to -0.5731 | Yes | |  |
| **Figure 7B p-CREB Protein**  One-way analysis of variance | |  | | |  |  |
| P value | | < 0.0001 | | |  |  |
| P value summary | | *** | | |  |  |
| Are means signif. different? (P < 0.05) | | Yes | | |  |  |
| Number of groups | | 3 | | |  |  |
| F | | 51.66 | | |  | |
| R squared | | 0.8742 | | |  |  |

| ANOVA Table | SS | df | MS |
| --- | --- | --- | --- |
| Treatment (between columns) | 0.9395 | 2 | 0.4698 |
| Residual (within columns) | 0.1364 | 15 | 0.009094 |
| Total | 1.076 | 17 |  |

| Dunnett's multiple comparisons test | Mean Diff. | 95.00% CI of diff. | Significant? |
| --- | --- | --- | --- |
|  |  |  |  |
| Stroke vs. Sham | -0.4022 | -0.5365 to -0.2679 | Yes |
| Stroke vs. Stroke+CBL | -0.5381 | -0.6724 to -0.4038 | Yes |
